# Supplementary figures and images for: Propofol induces mitochondrial-associated protein LRPPRC and protects mitochondria against hypoxia in cardiac cells
Source: PLoS One. 2020 Sep 8;15(9):e0238857. doi: 10.1371/journal.pone.0238857 (PMC7478836; doi:10.1371/journal.pone.0238857)

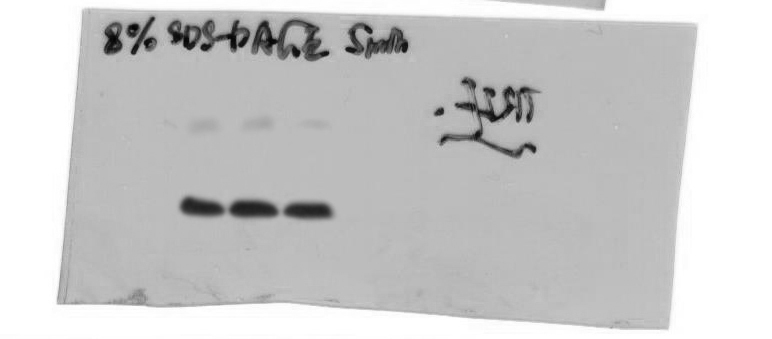

Supplement: S1 File — (ZIP) [file pone.0238857.s001.zip › original uncropped and unadjusted images/figure 1c/actin, caspase 8.jpg]

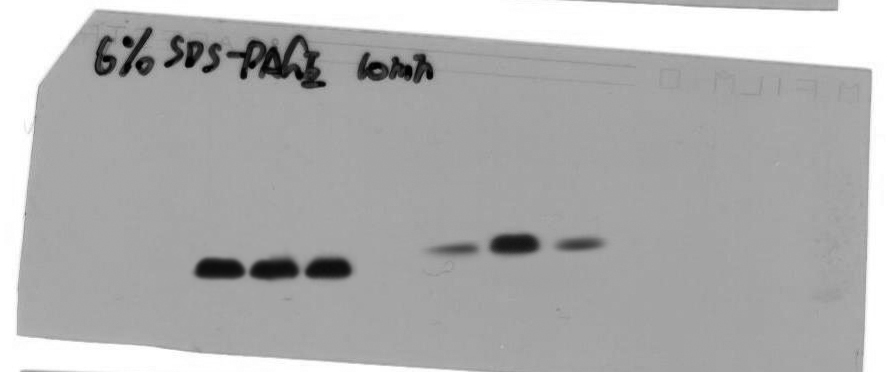

Supplement: S1 File — (ZIP) [file pone.0238857.s001.zip › original uncropped and unadjusted images/figure 1c/actin, caspase-cleaved.jpg]

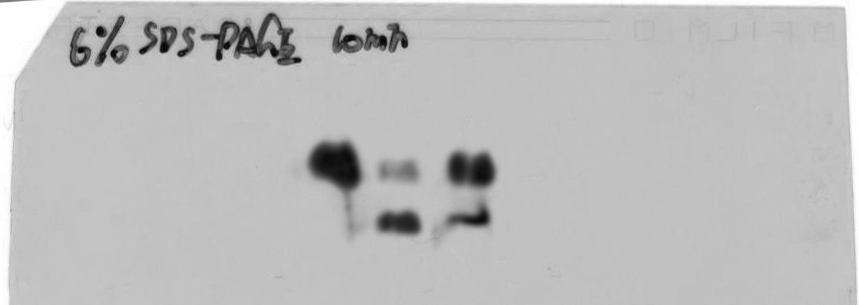

Supplement: S1 File — (ZIP) [file pone.0238857.s001.zip › original uncropped and unadjusted images/figure 1c/caspase-9.jpg]

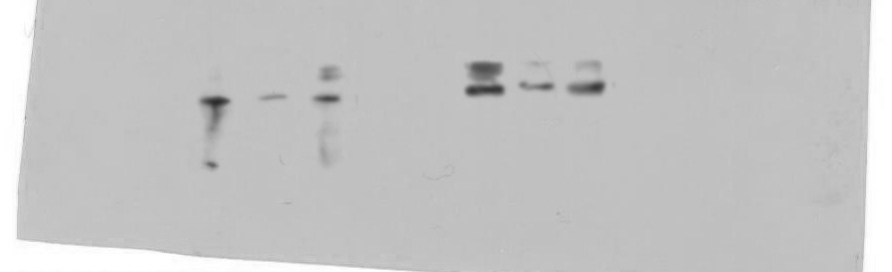

Supplement: S1 File — (ZIP) [file pone.0238857.s001.zip › original uncropped and unadjusted images/figure 1c/pro-caspase 9, cleaved-caspase 9.jpg]

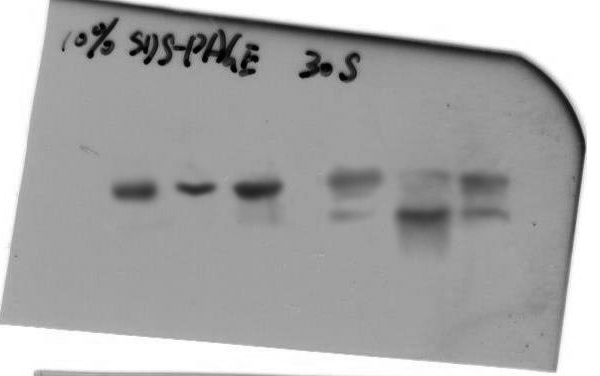

Supplement: S1 File — (ZIP) [file pone.0238857.s001.zip › original uncropped and unadjusted images/figure 1c/pro-caspase3, PRPP.jpg]

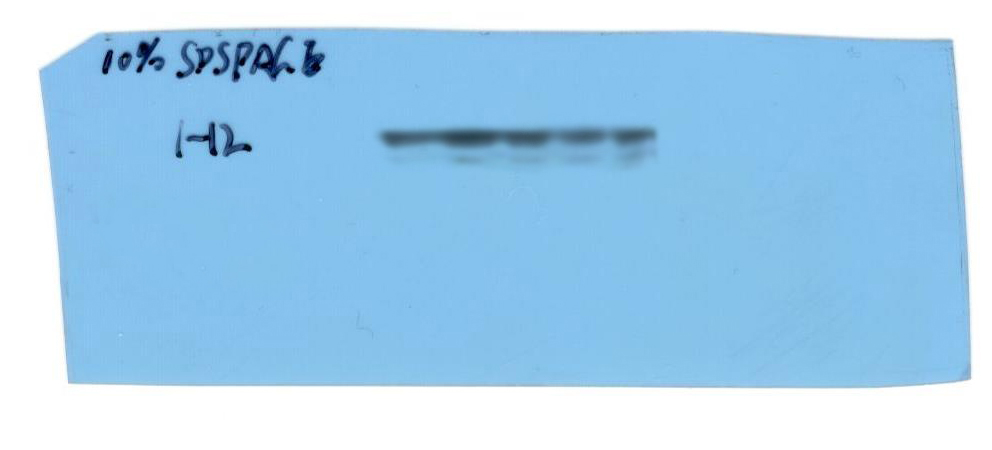

Supplement: S1 File — (ZIP) [file pone.0238857.s001.zip › original uncropped and unadjusted images/figure 2a/Beclin 1.jpg]

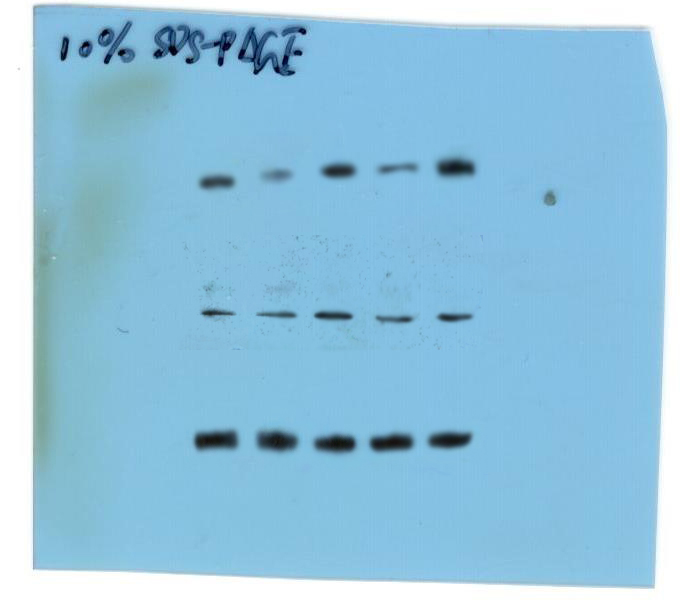

Supplement: S1 File — (ZIP) [file pone.0238857.s001.zip › original uncropped and unadjusted images/figure 2a/LRPPRC, BCL2, ACTIN.jpg]

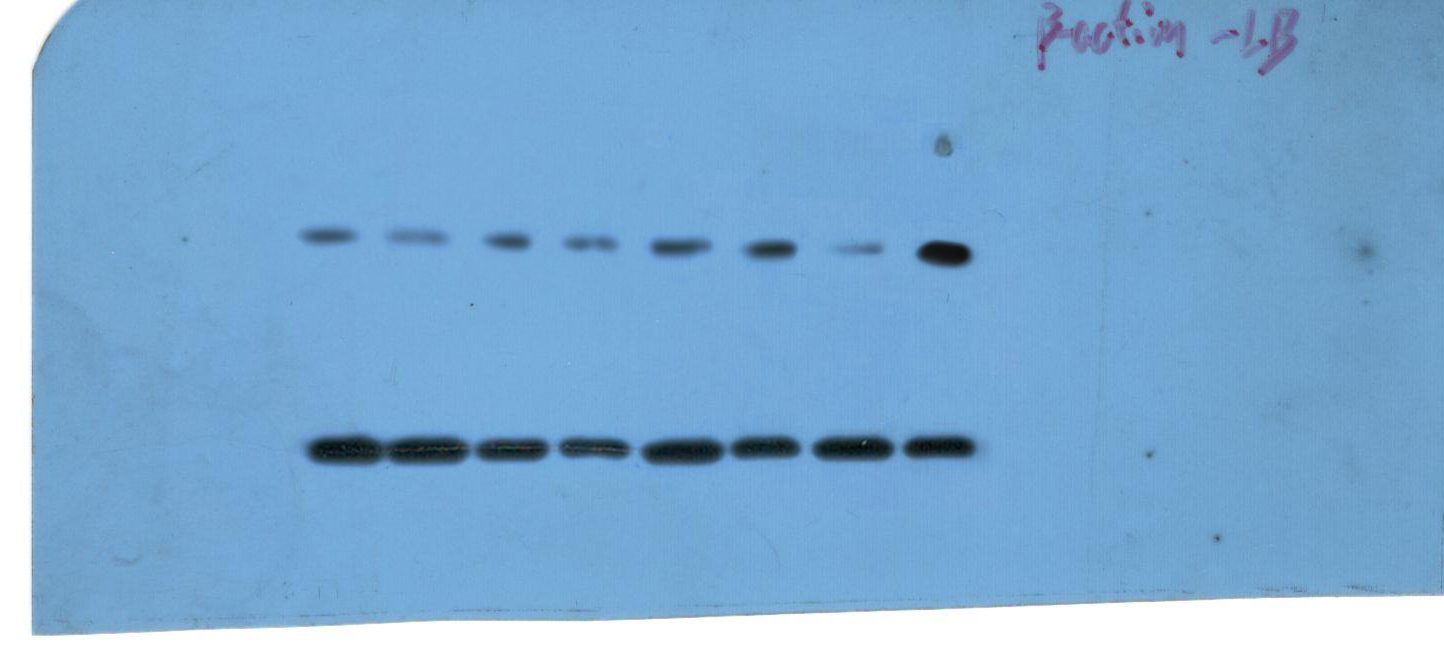

Supplement: S1 File — (ZIP) [file pone.0238857.s001.zip › original uncropped and unadjusted images/figure 2d/f2d.jpg]

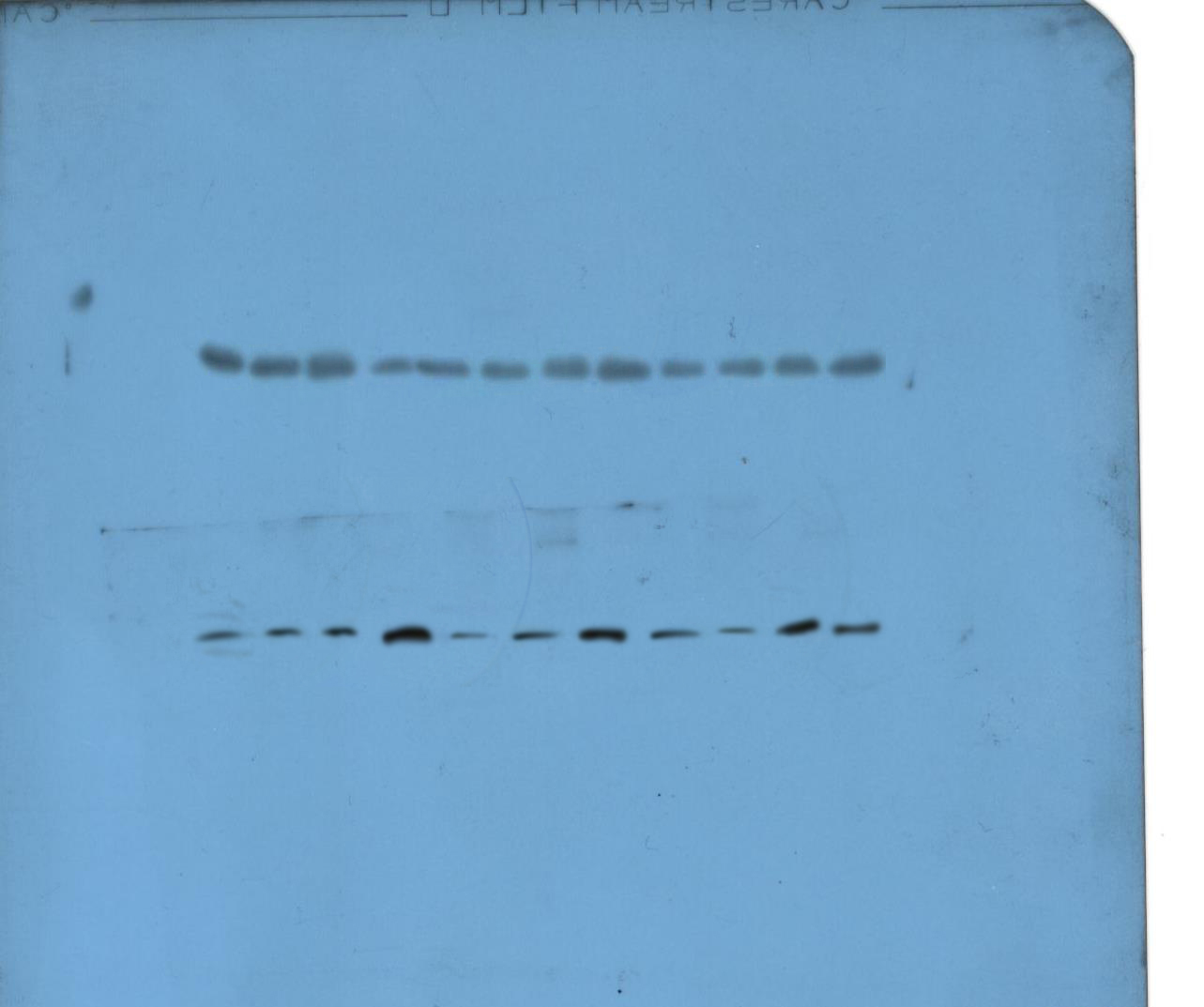

Supplement: S1 File — (ZIP) [file pone.0238857.s001.zip › original uncropped and unadjusted images/figure 3a/actin, lrpprc.jpg]

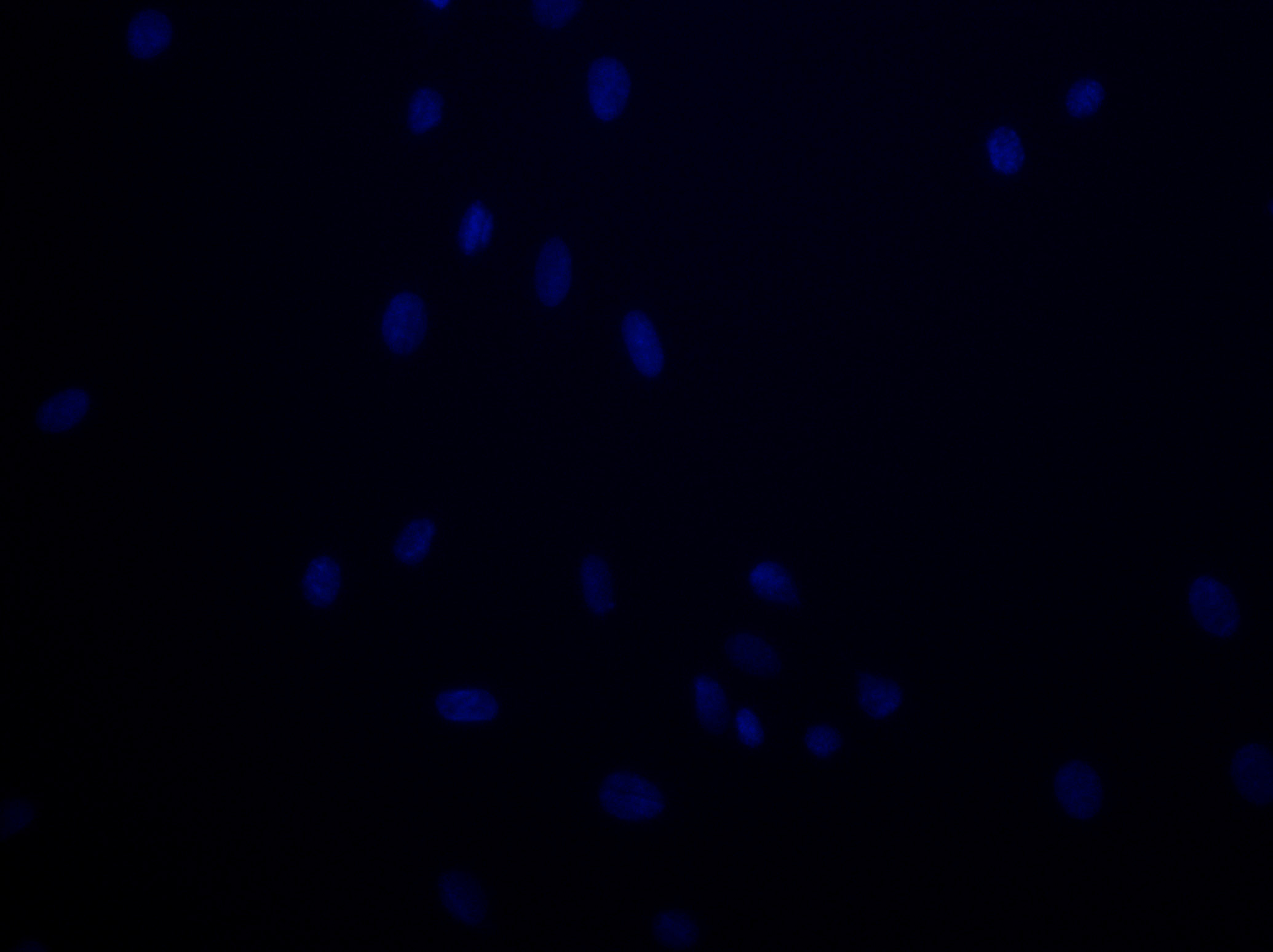

Supplement: S1 File — (ZIP) [file pone.0238857.s001.zip › original uncropped and unadjusted images/figure 4a/1-5 B.jpg]

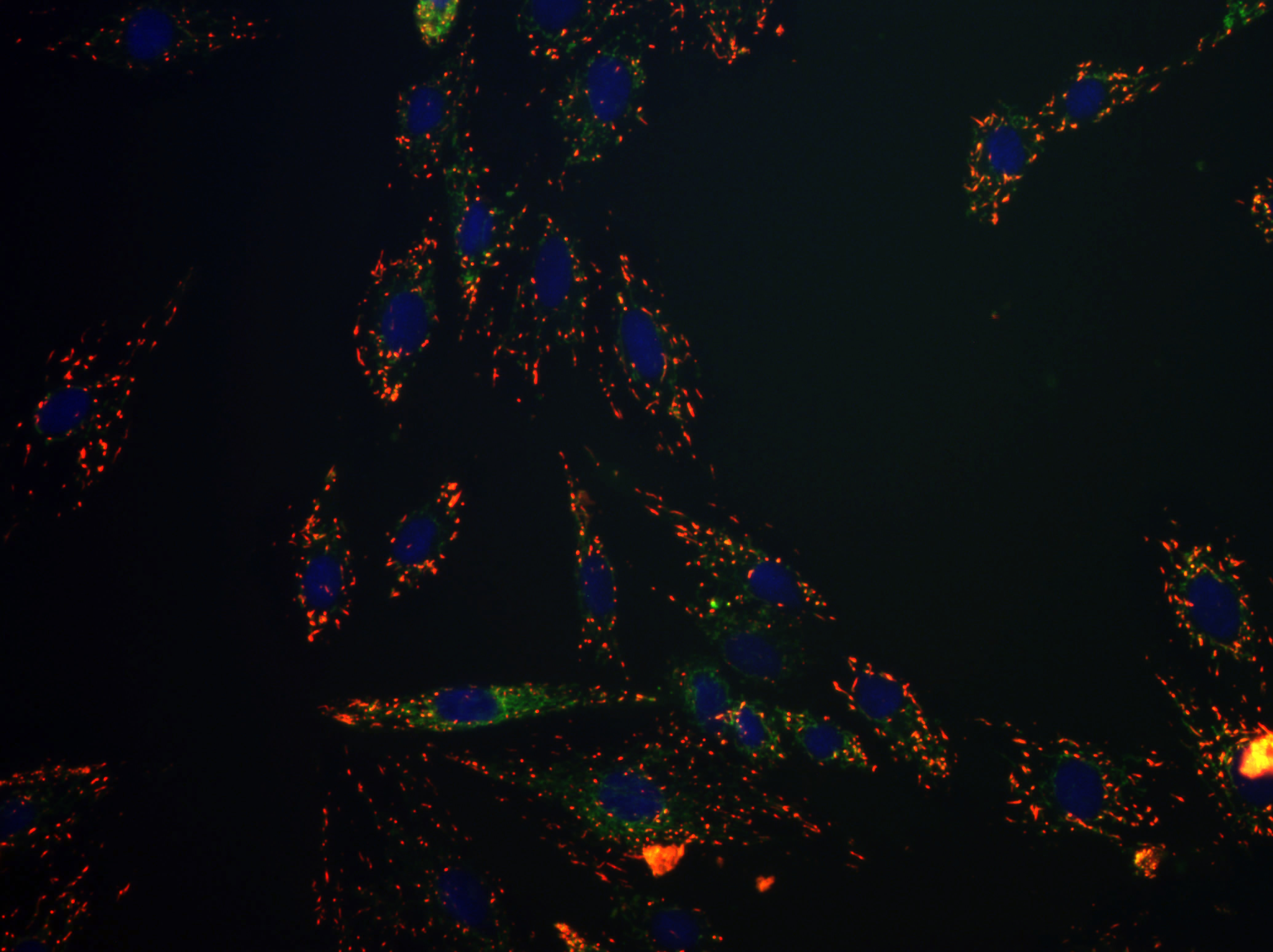

Supplement: S1 File — (ZIP) [file pone.0238857.s001.zip › original uncropped and unadjusted images/figure 4a/1-5 G -MERGED.jpg]

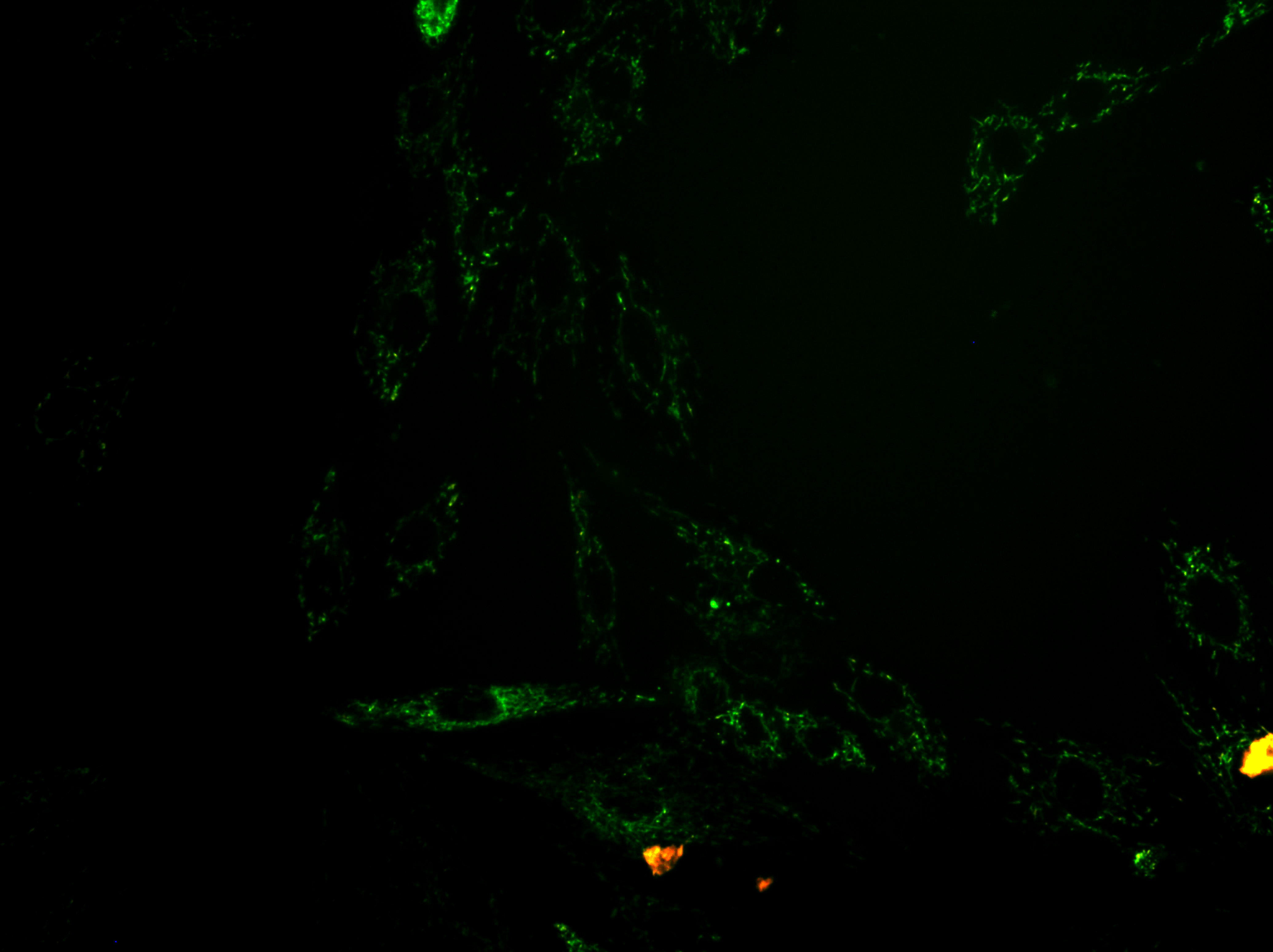

Supplement: S1 File — (ZIP) [file pone.0238857.s001.zip › original uncropped and unadjusted images/figure 4a/1-5 G.jpg]

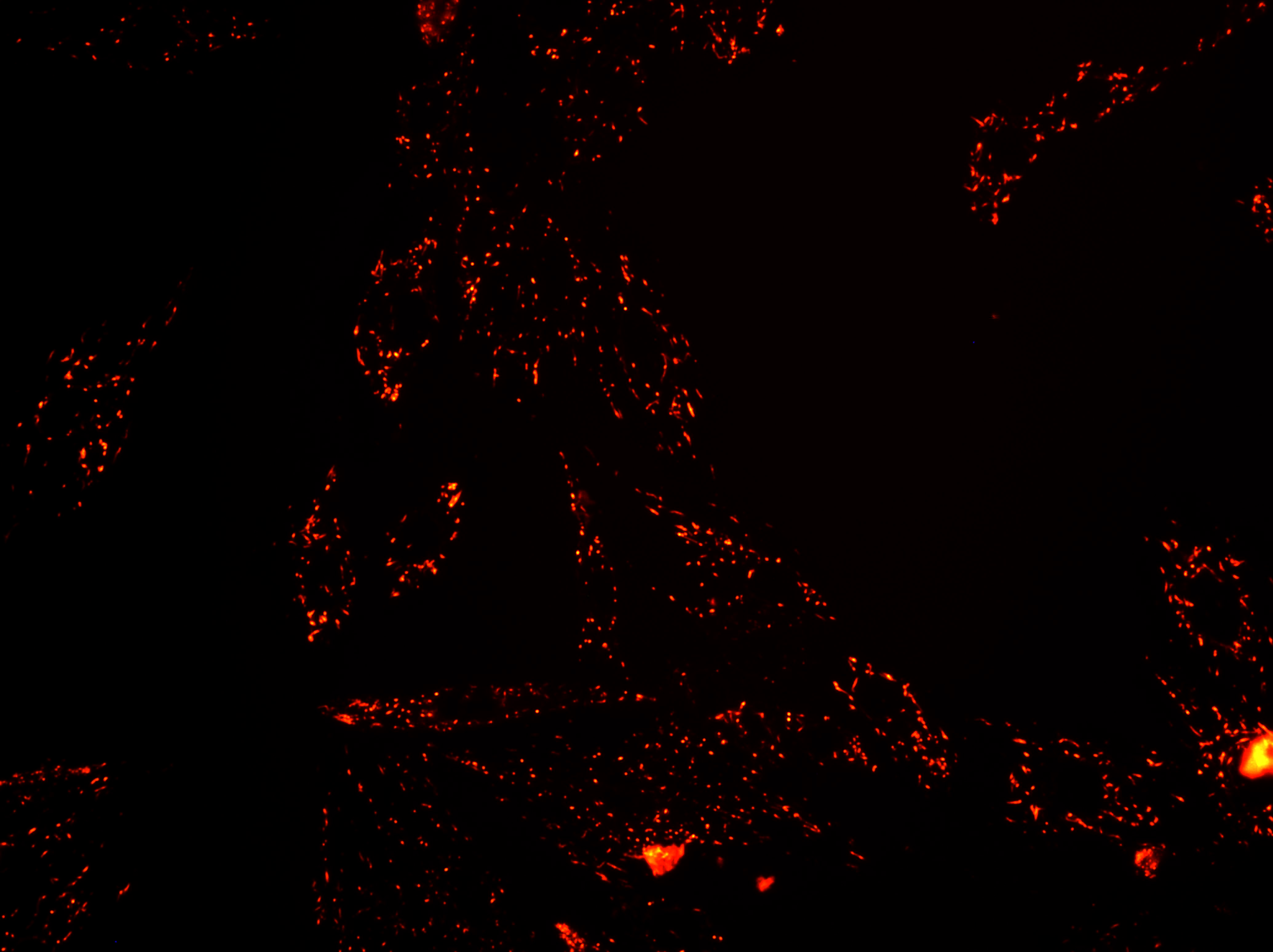

Supplement: S1 File — (ZIP) [file pone.0238857.s001.zip › original uncropped and unadjusted images/figure 4a/1-5 R.jpg]

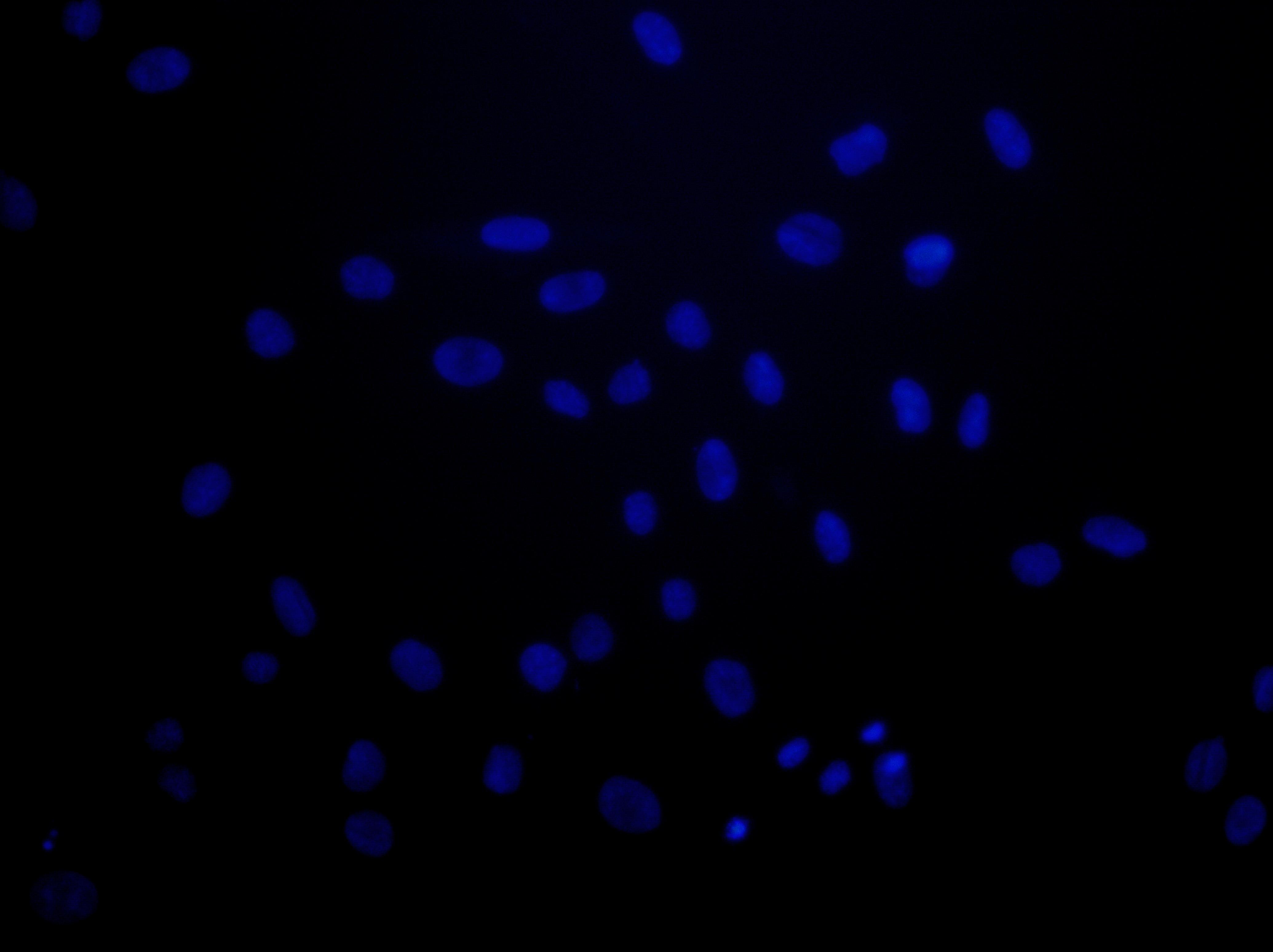

Supplement: S1 File — (ZIP) [file pone.0238857.s001.zip › original uncropped and unadjusted images/figure 4a/17B.jpg]

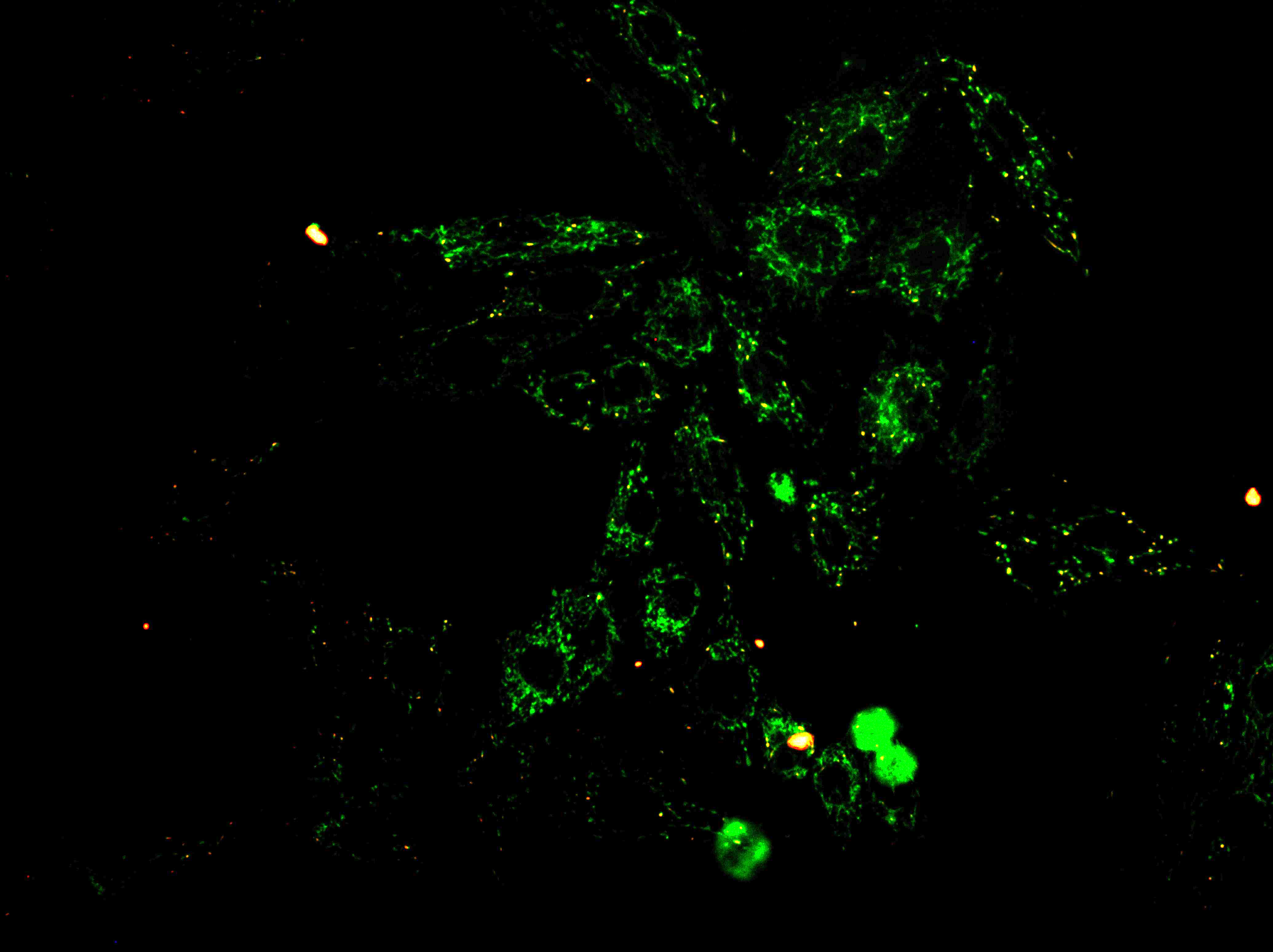

Supplement: S1 File — (ZIP) [file pone.0238857.s001.zip › original uncropped and unadjusted images/figure 4a/17G.jpg]

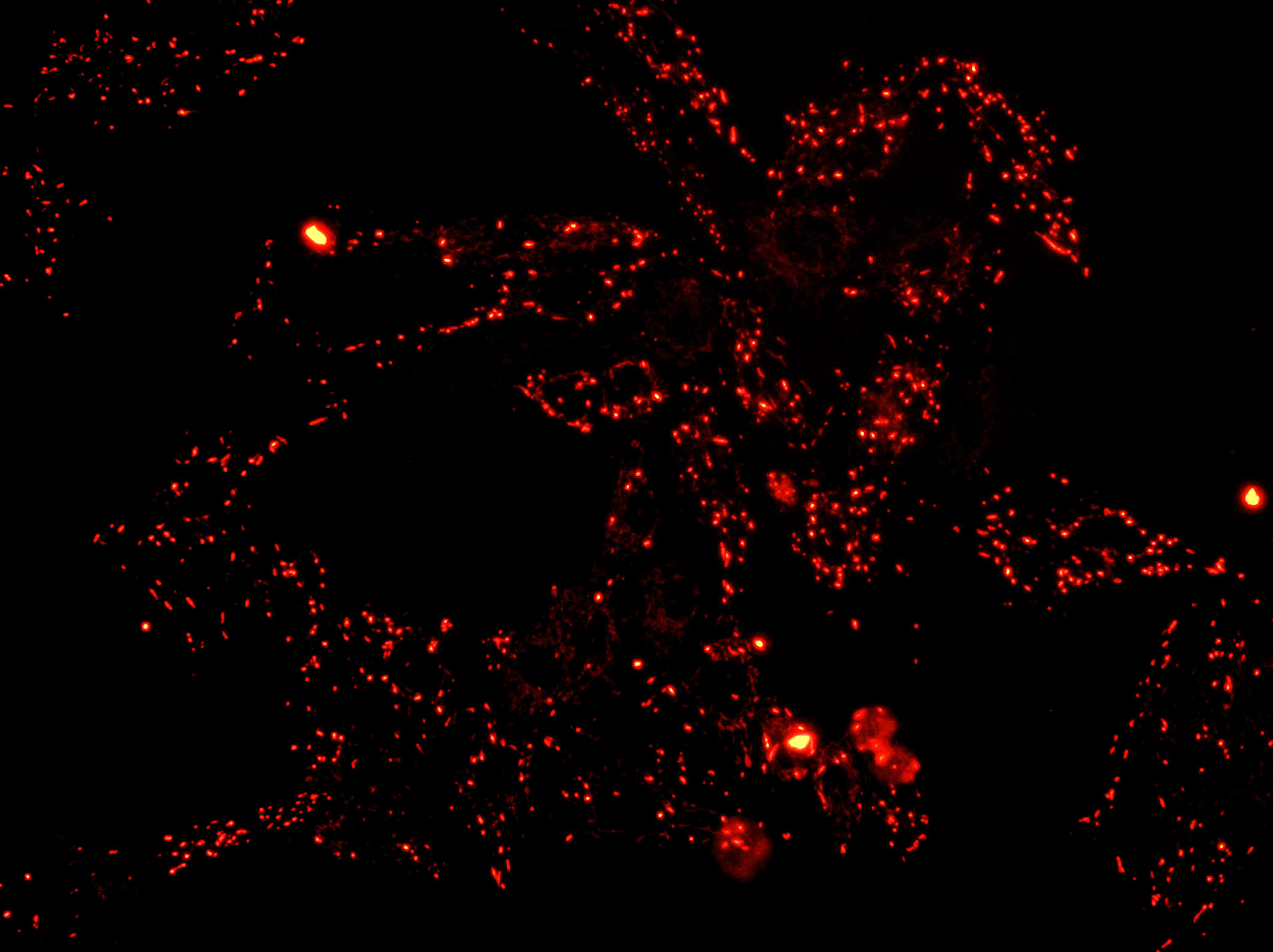

Supplement: S1 File — (ZIP) [file pone.0238857.s001.zip › original uncropped and unadjusted images/figure 4a/17R.jpg]

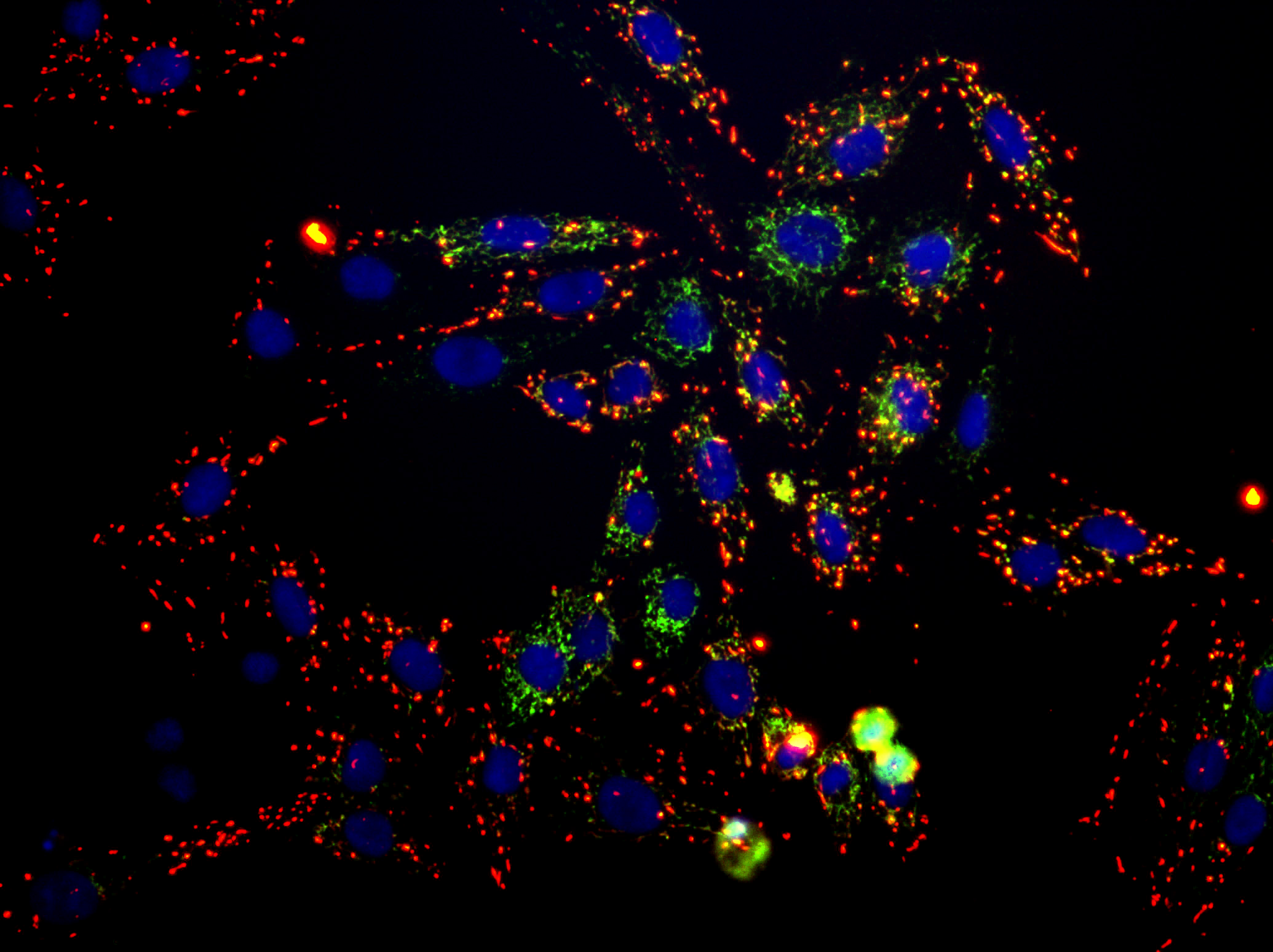

Supplement: S1 File — (ZIP) [file pone.0238857.s001.zip › original uncropped and unadjusted images/figure 4a/17m.jpg]

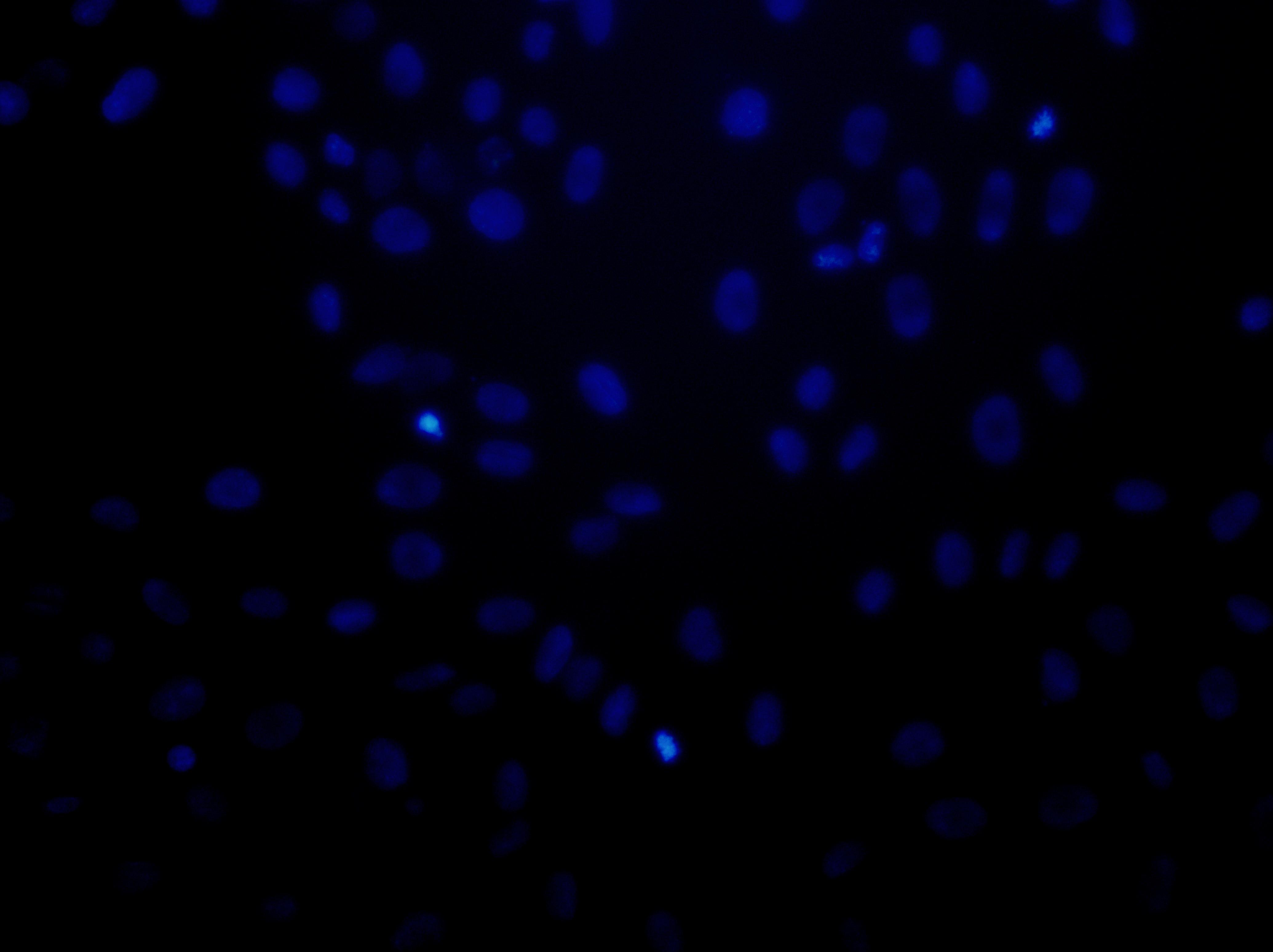

Supplement: S1 File — (ZIP) [file pone.0238857.s001.zip › original uncropped and unadjusted images/figure 4a/7B.jpg]

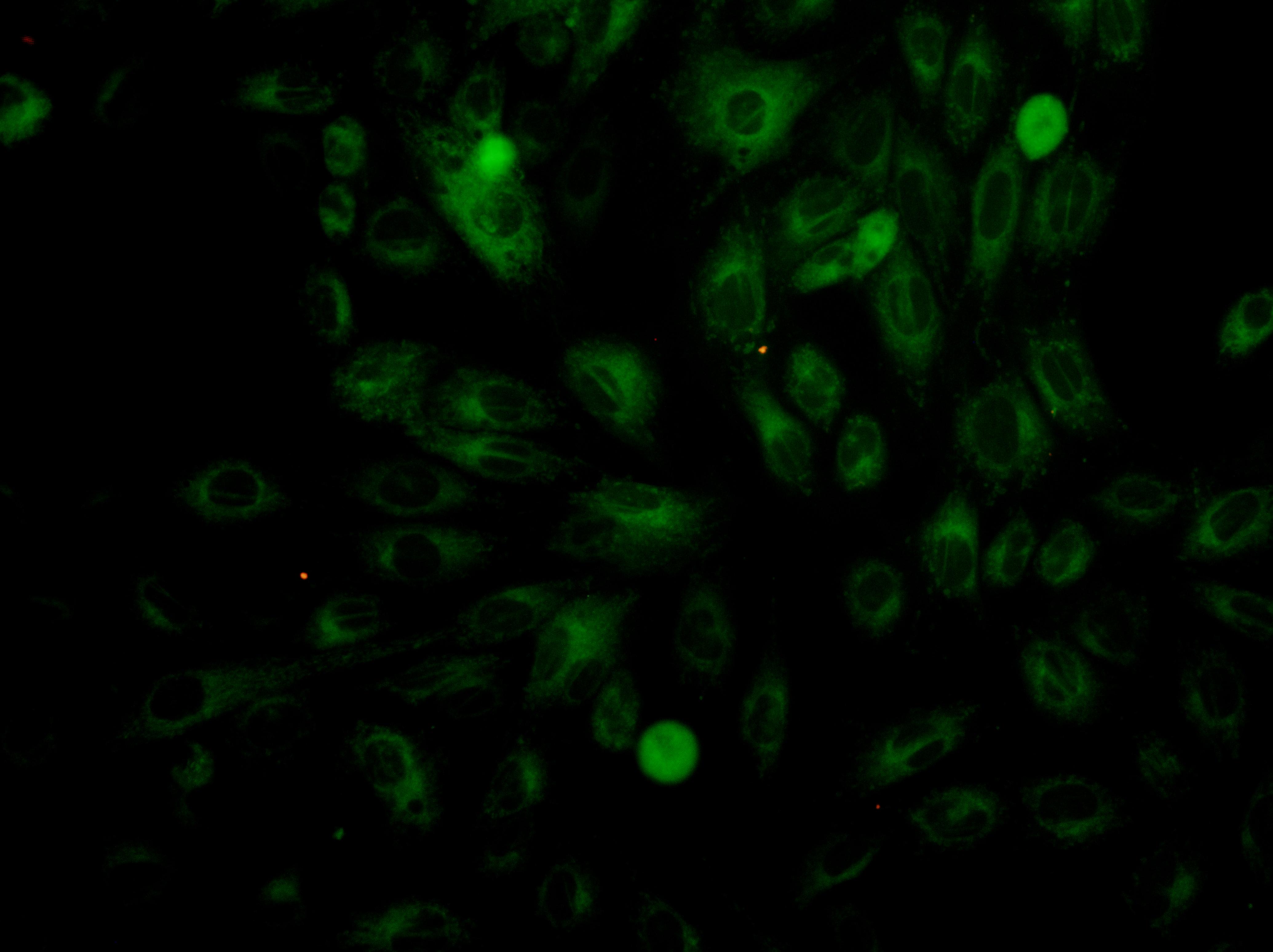

Supplement: S1 File — (ZIP) [file pone.0238857.s001.zip › original uncropped and unadjusted images/figure 4a/7G.jpg]

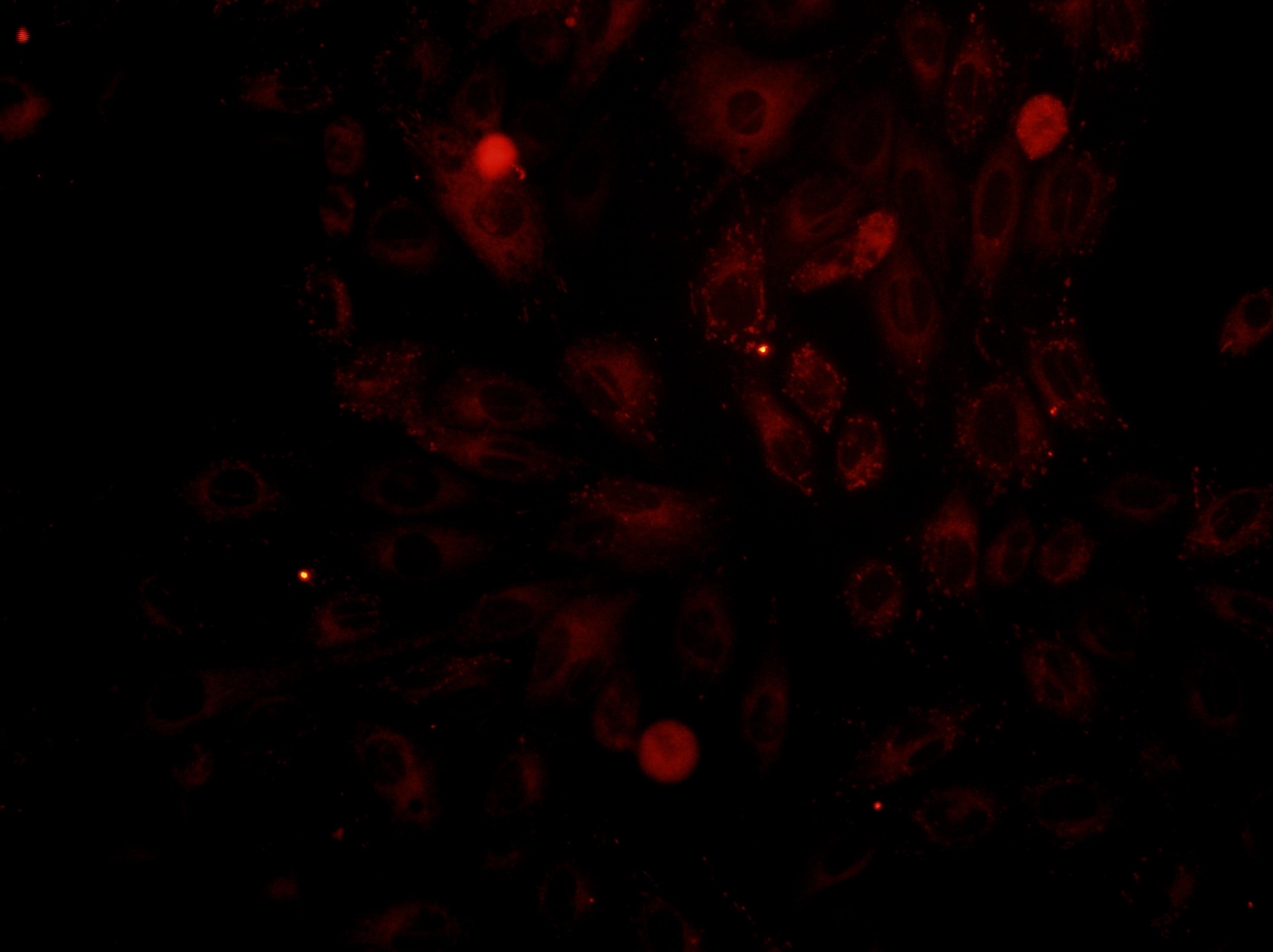

Supplement: S1 File — (ZIP) [file pone.0238857.s001.zip › original uncropped and unadjusted images/figure 4a/7R.jpg]

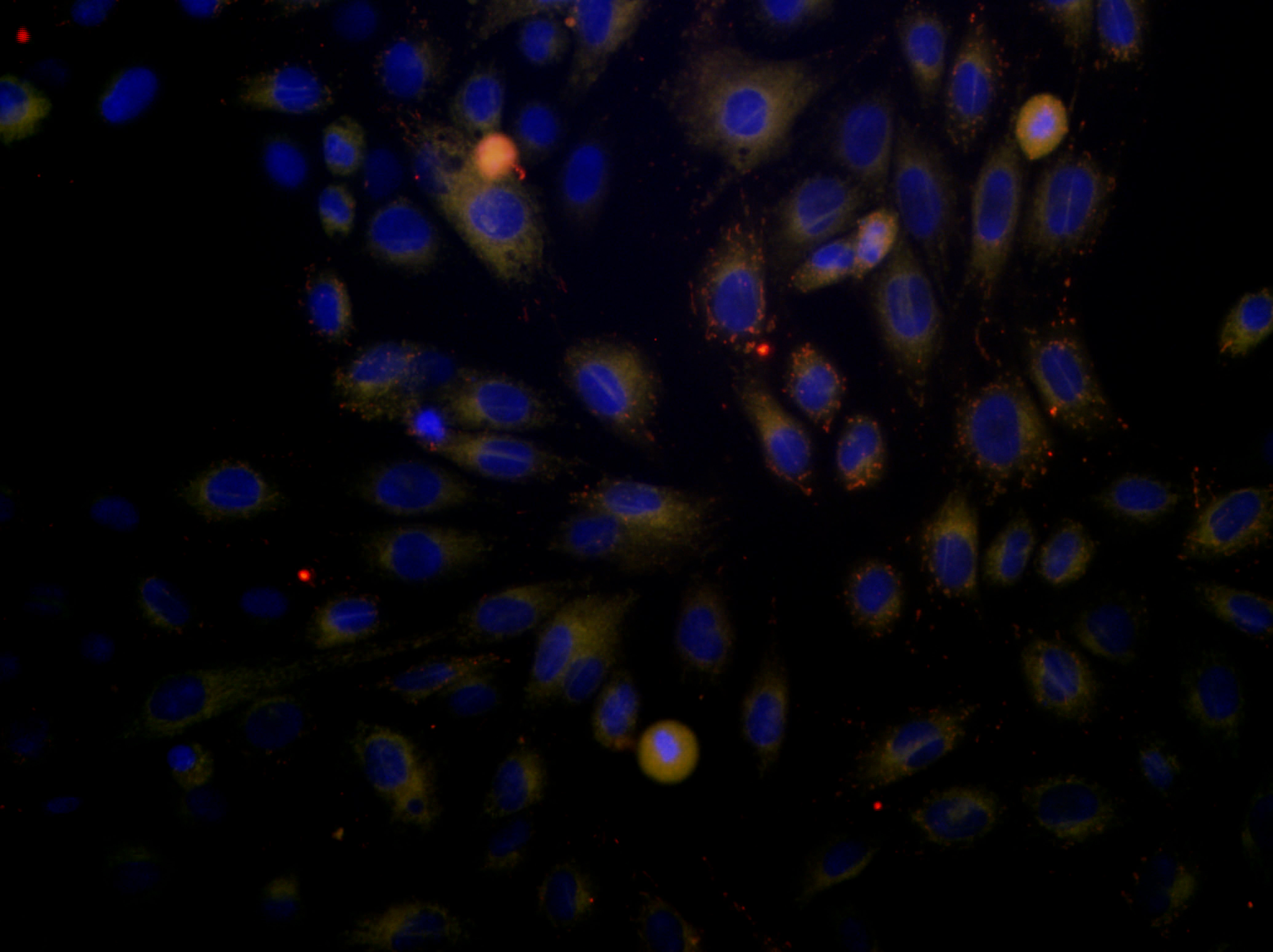

Supplement: S1 File — (ZIP) [file pone.0238857.s001.zip › original uncropped and unadjusted images/figure 4a/7m.jpg]

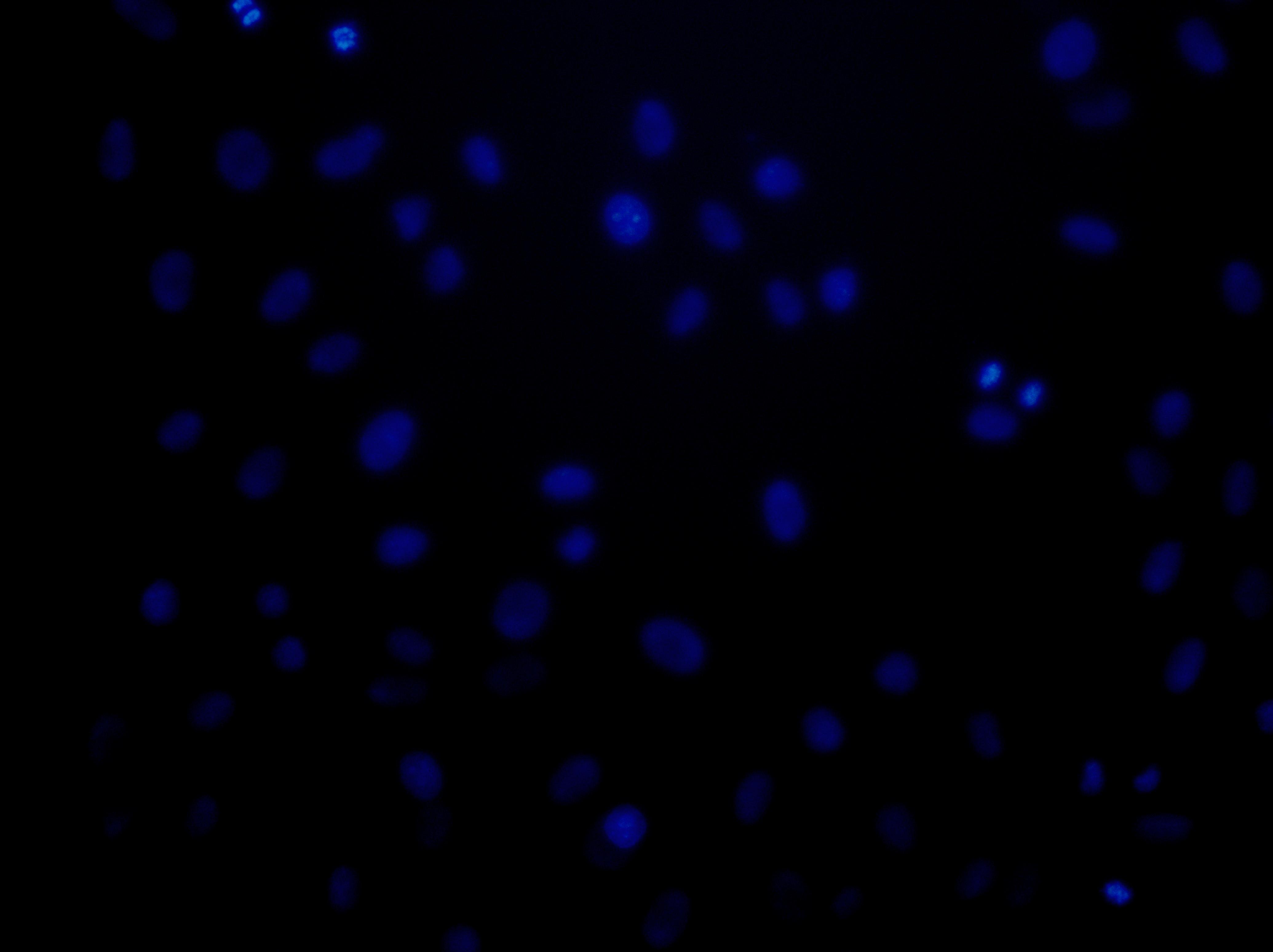

Supplement: S1 File — (ZIP) [file pone.0238857.s001.zip › original uncropped and unadjusted images/figure 4a/8B.jpg]

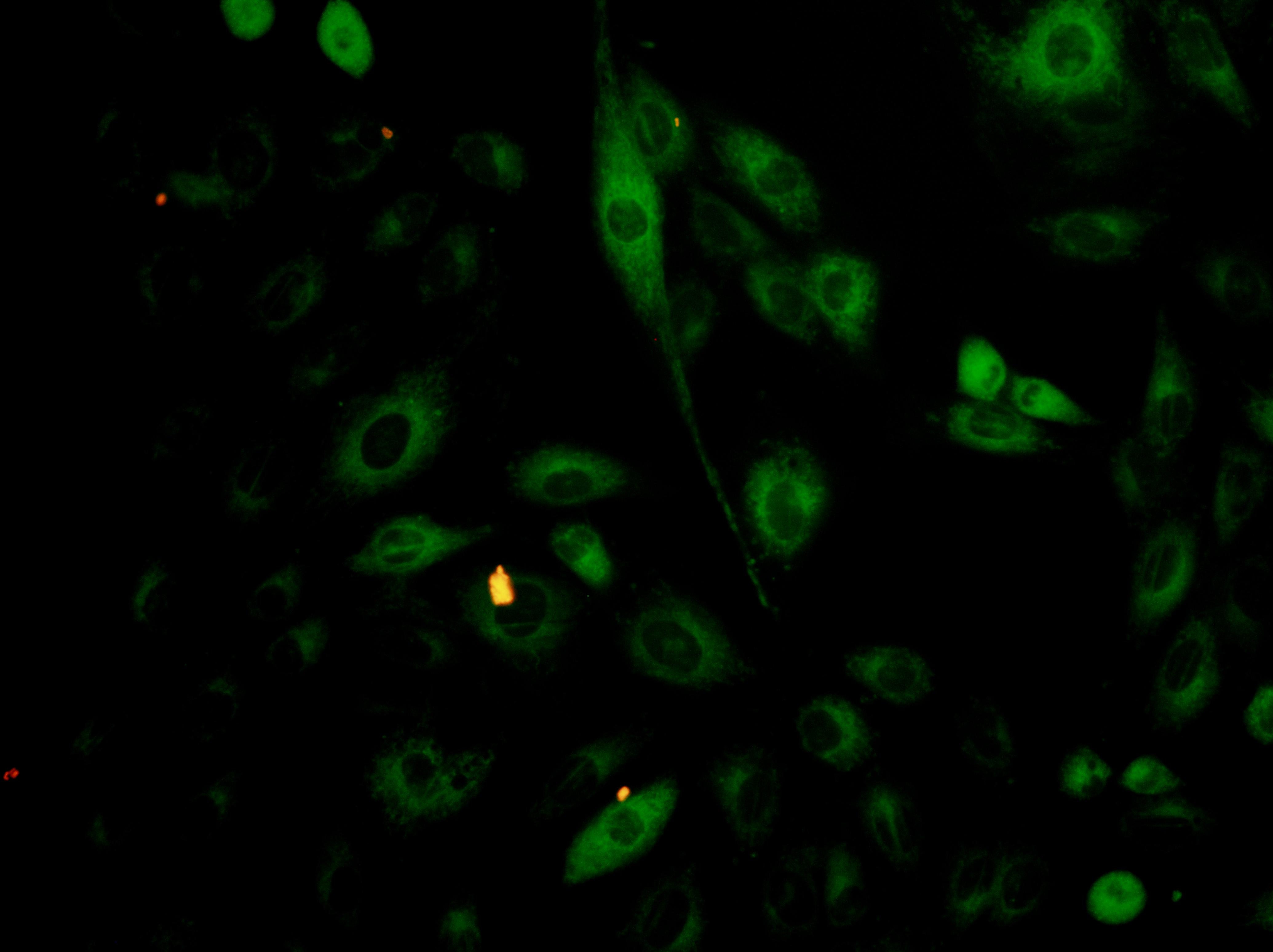

Supplement: S1 File — (ZIP) [file pone.0238857.s001.zip › original uncropped and unadjusted images/figure 4a/8G.jpg]

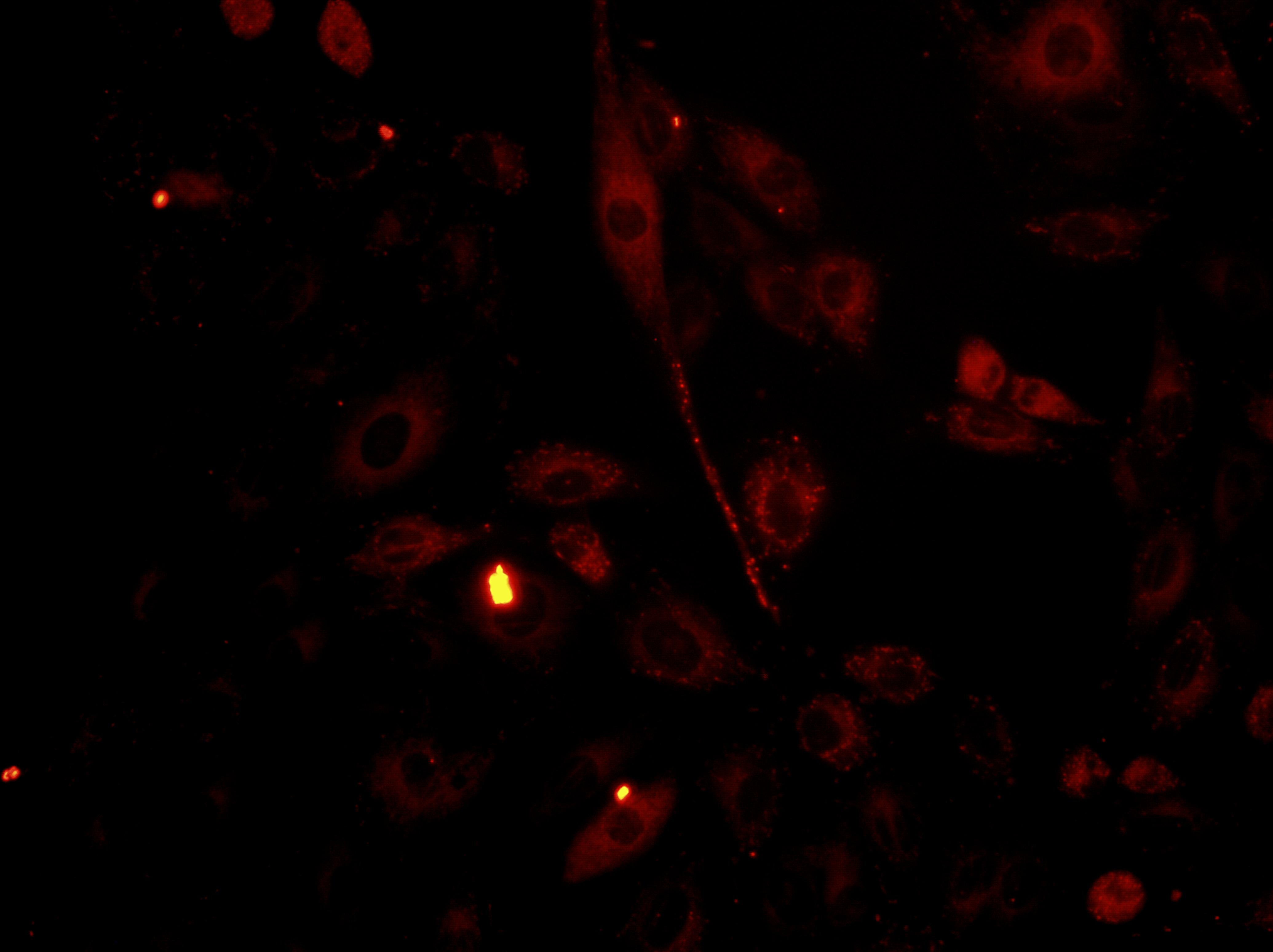

Supplement: S1 File — (ZIP) [file pone.0238857.s001.zip › original uncropped and unadjusted images/figure 4a/8R.jpg]

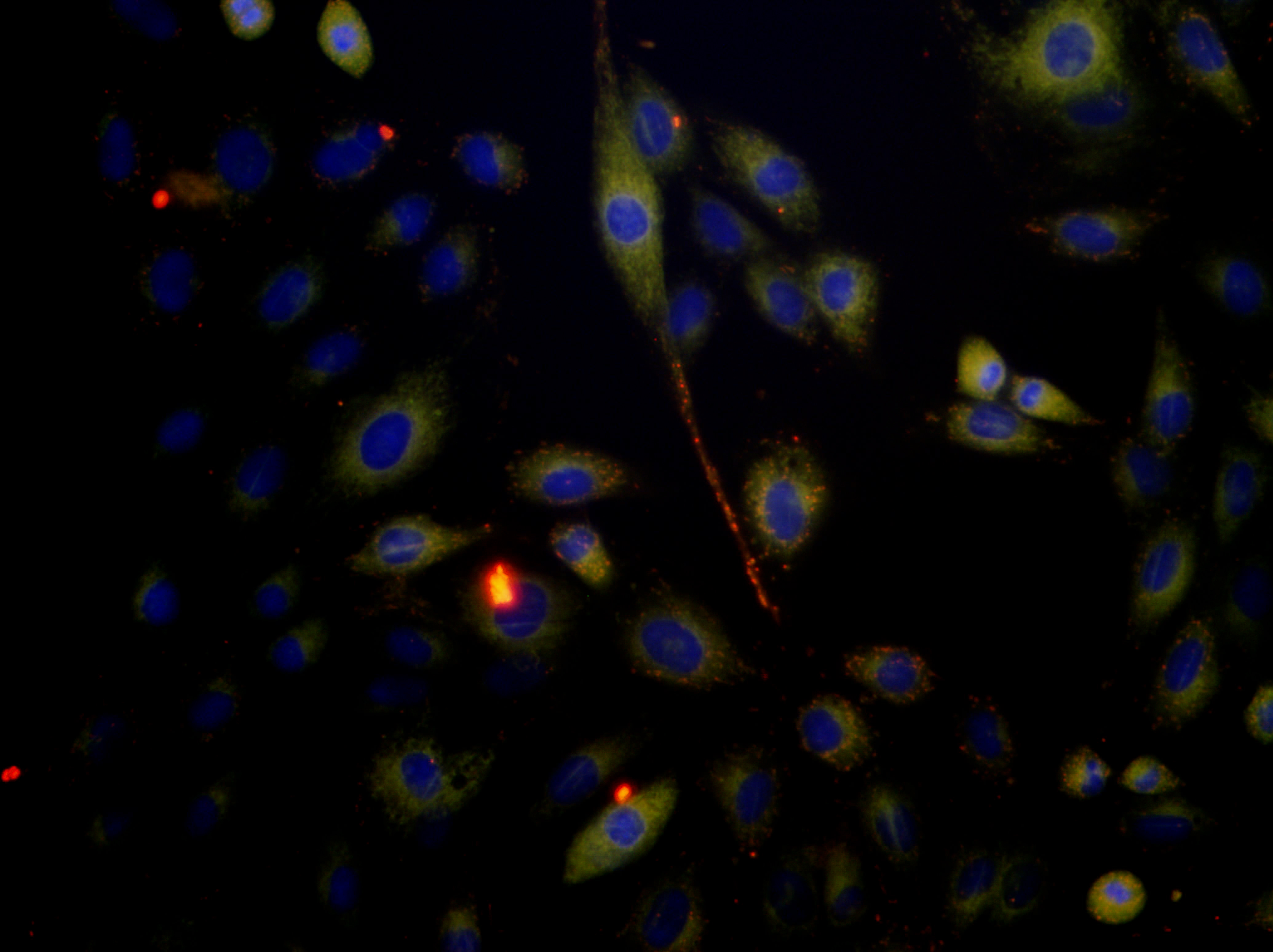

Supplement: S1 File — (ZIP) [file pone.0238857.s001.zip › original uncropped and unadjusted images/figure 4a/8m.jpg]

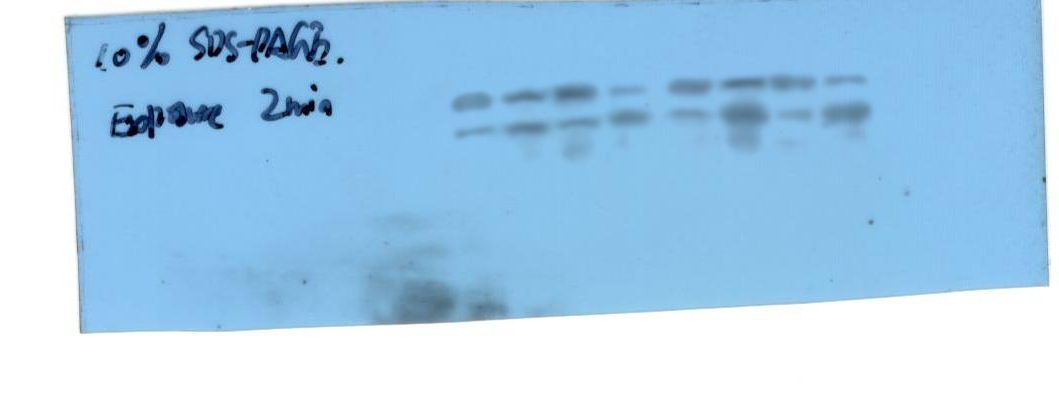

Supplement: S1 File — (ZIP) [file pone.0238857.s001.zip › original uncropped and unadjusted images/figure 4e/LC3.jpg]

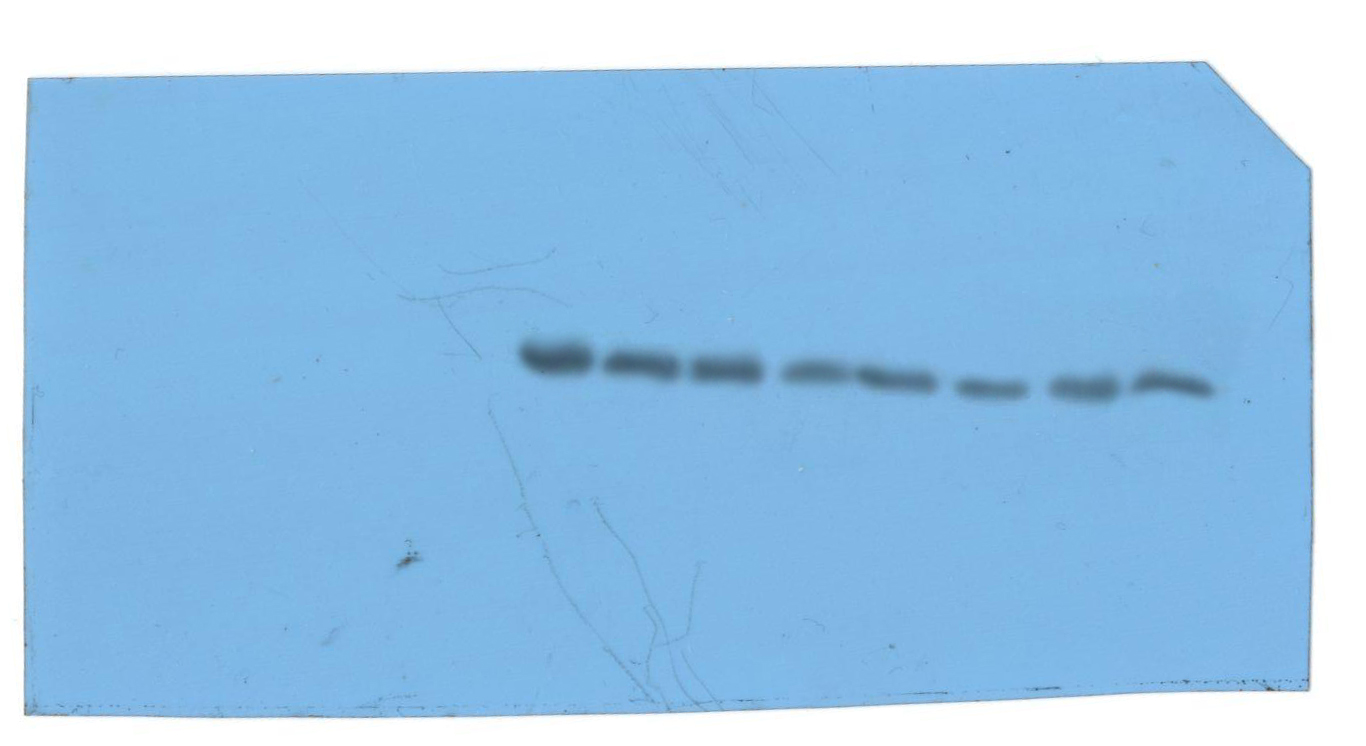

Supplement: S1 File — (ZIP) [file pone.0238857.s001.zip › original uncropped and unadjusted images/figure 4e/actin.jpg]

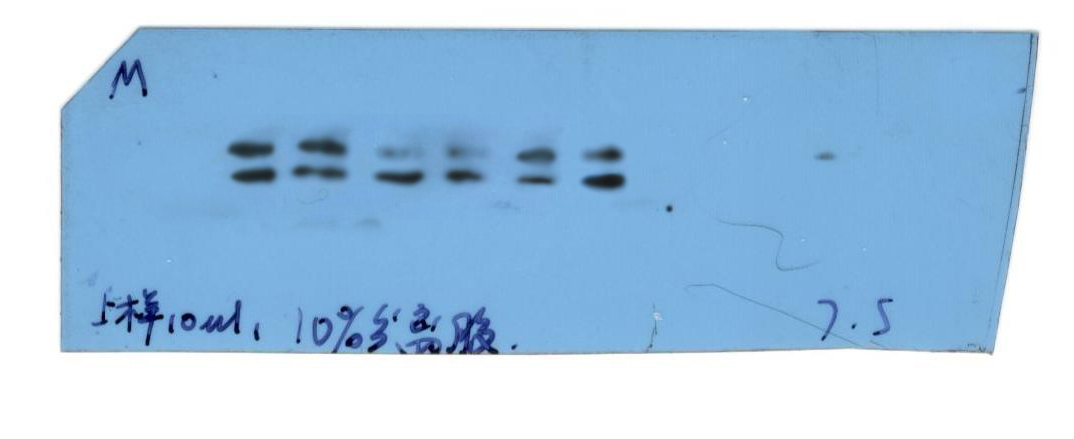

Supplement: S1 File — (ZIP) [file pone.0238857.s001.zip › original uncropped and unadjusted images/figure 5a/LC3B.jpg]

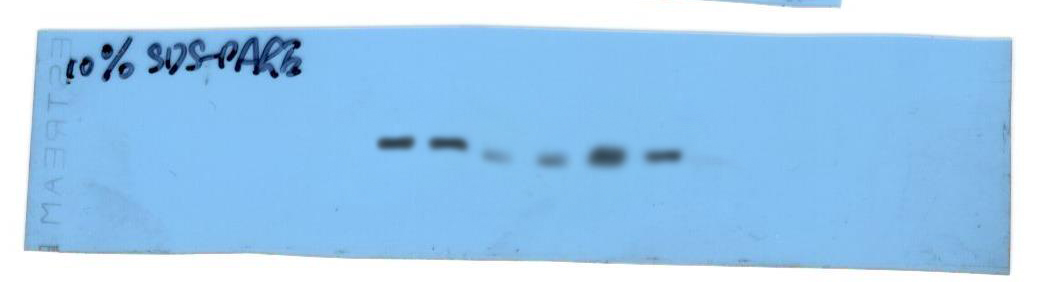

Supplement: S1 File — (ZIP) [file pone.0238857.s001.zip › original uncropped and unadjusted images/figure 5a/LRPPRC.jpg]

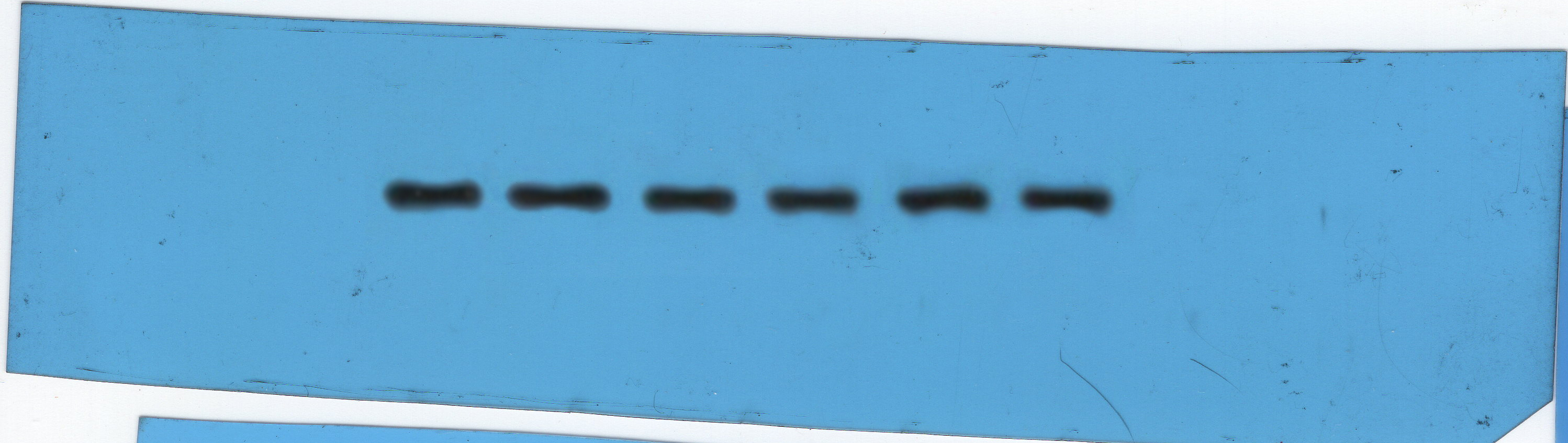

Supplement: S1 File — (ZIP) [file pone.0238857.s001.zip › original uncropped and unadjusted images/figure 5a/actin.jpg]

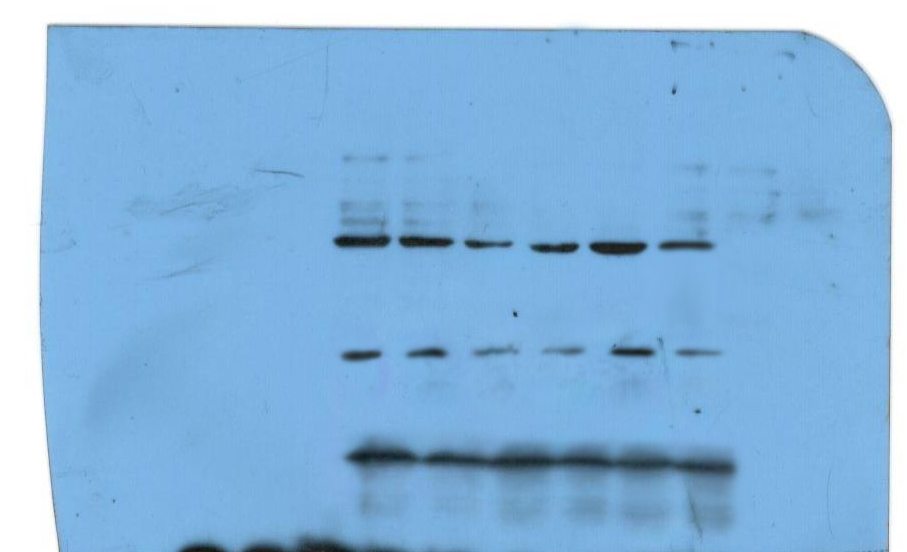

Supplement: S1 File — (ZIP) [file pone.0238857.s001.zip › original uncropped and unadjusted images/figure 6a/LRPPRC, Bcl-2, Beclin 1.jpg]

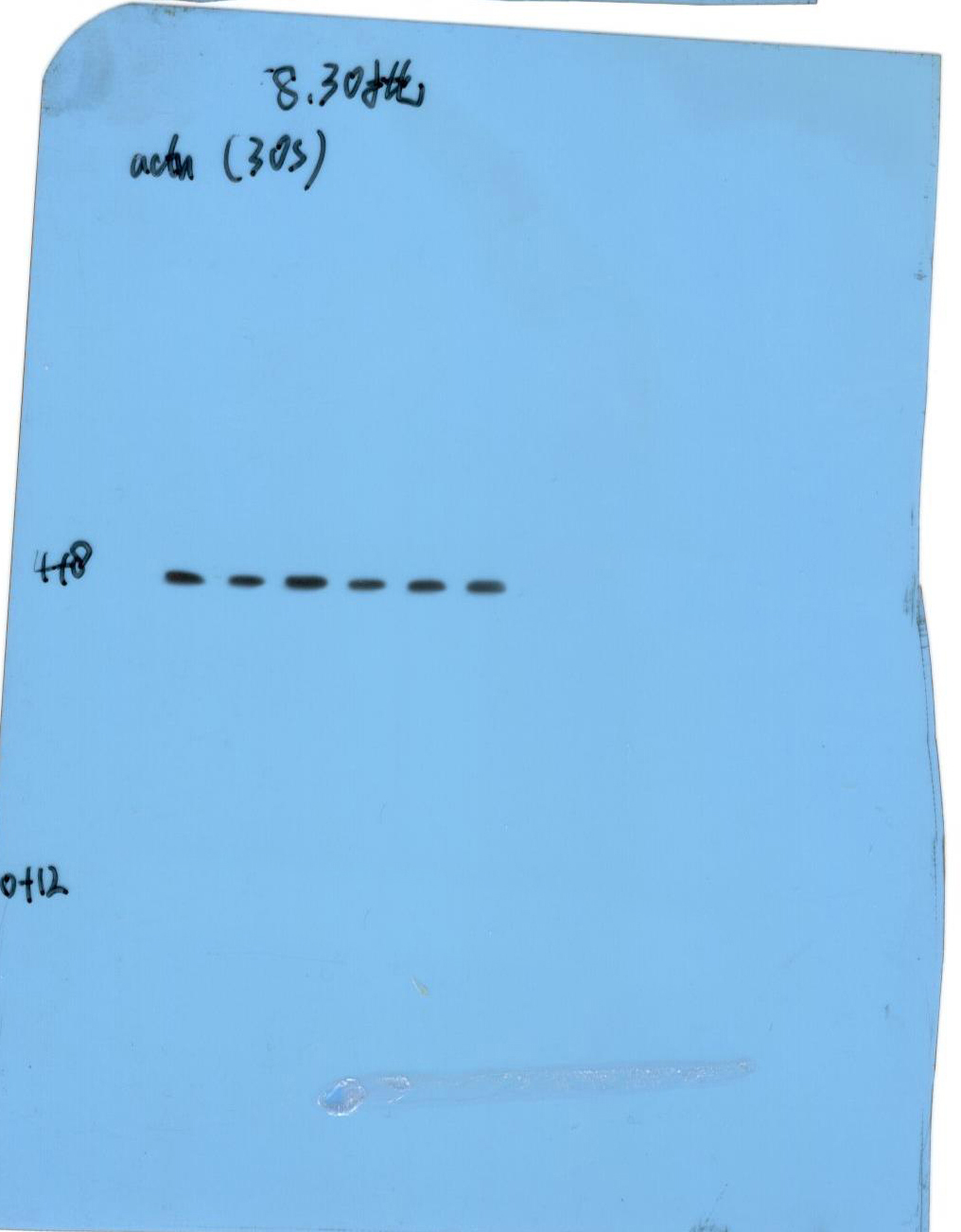

Supplement: S1 File — (ZIP) [file pone.0238857.s001.zip › original uncropped and unadjusted images/figure 6a/actin.jpg]

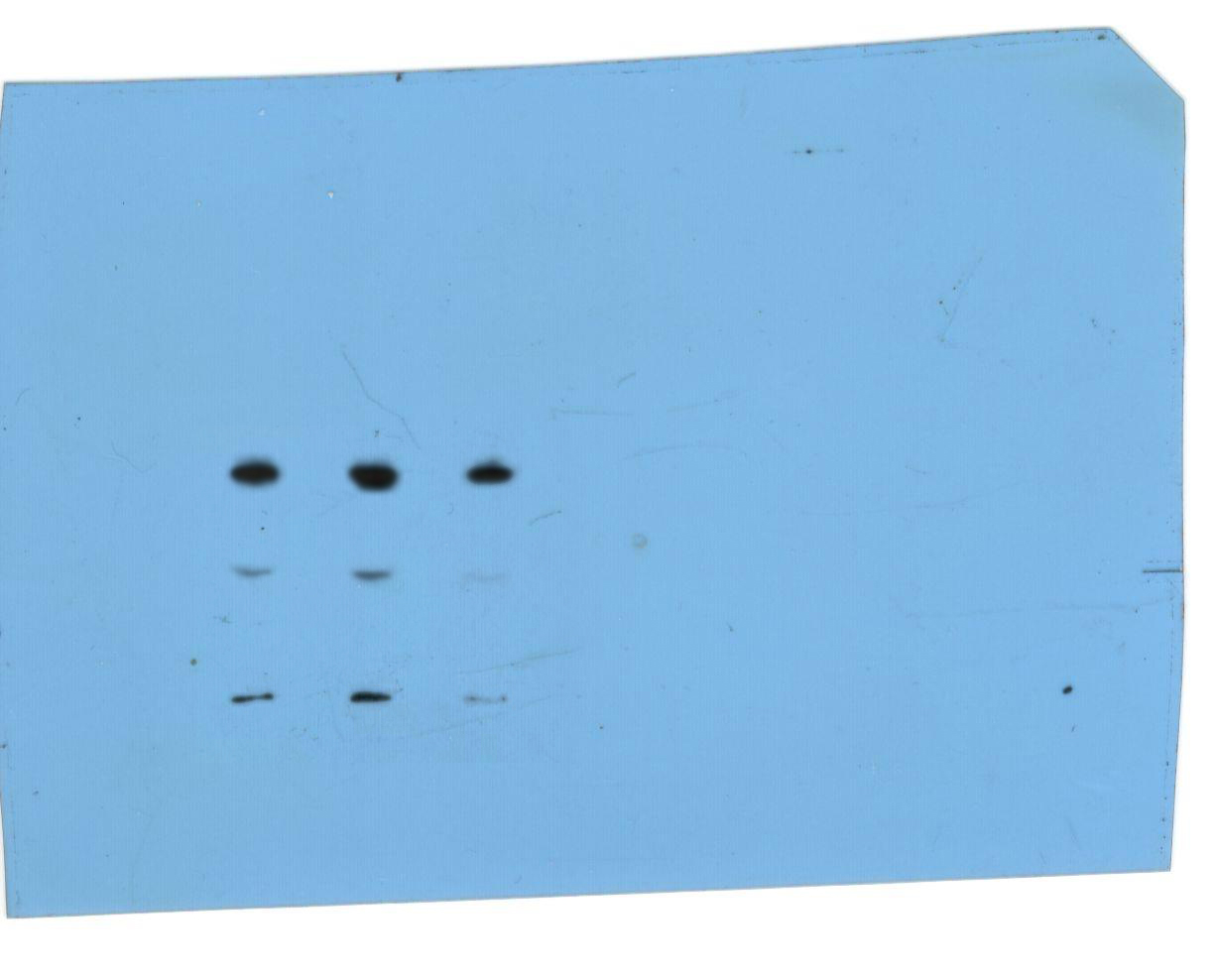

Supplement: S1 File — (ZIP) [file pone.0238857.s001.zip › original uncropped and unadjusted images/figure 6b/IP-2.jpg]

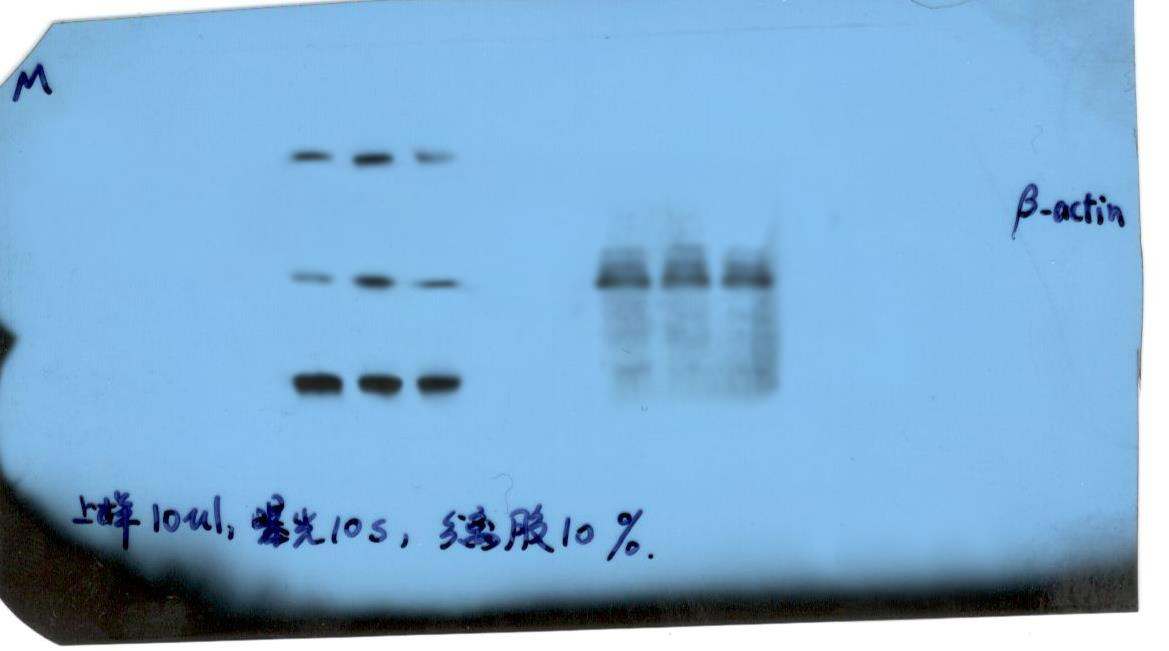

Supplement: S1 File — (ZIP) [file pone.0238857.s001.zip › original uncropped and unadjusted images/figure 6b/Input.jpg]

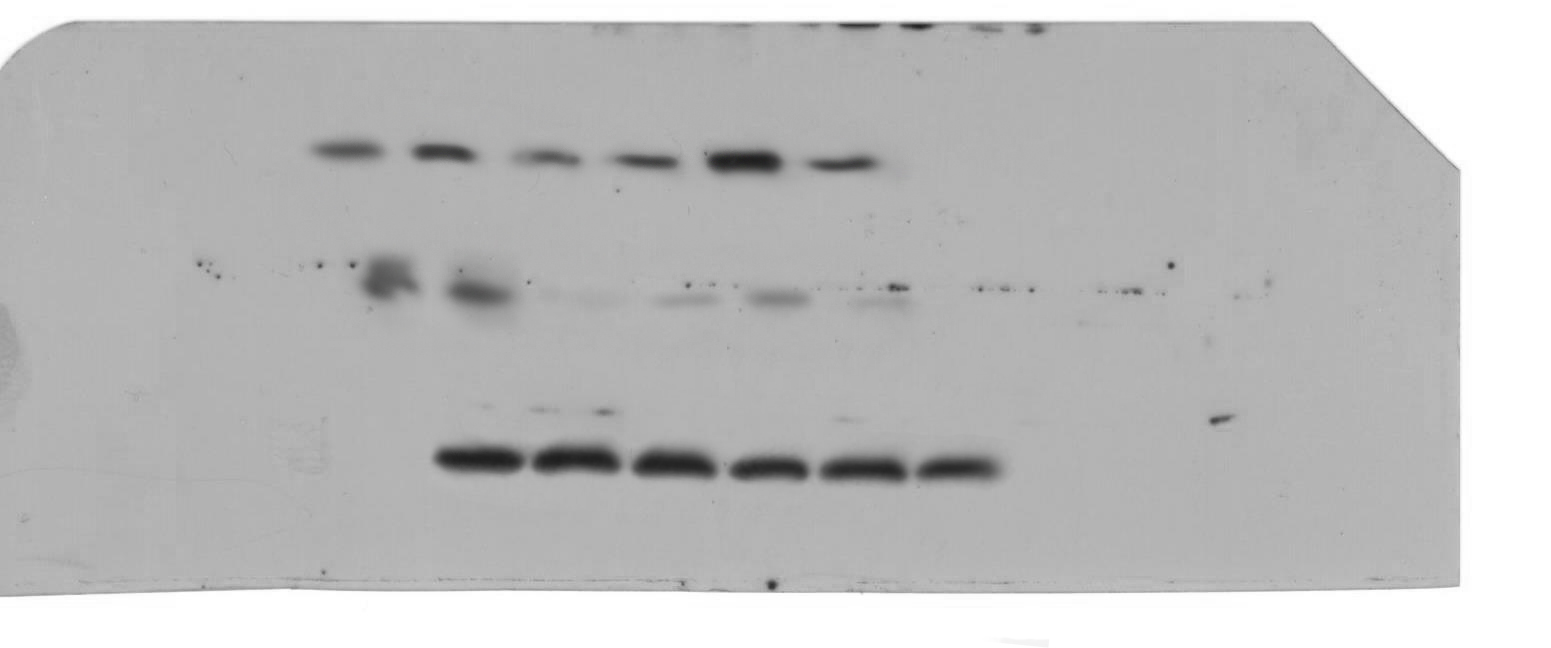

Supplement: S1 File — (ZIP) [file pone.0238857.s001.zip › original uncropped and unadjusted images/figure 7a/wb.jpg]

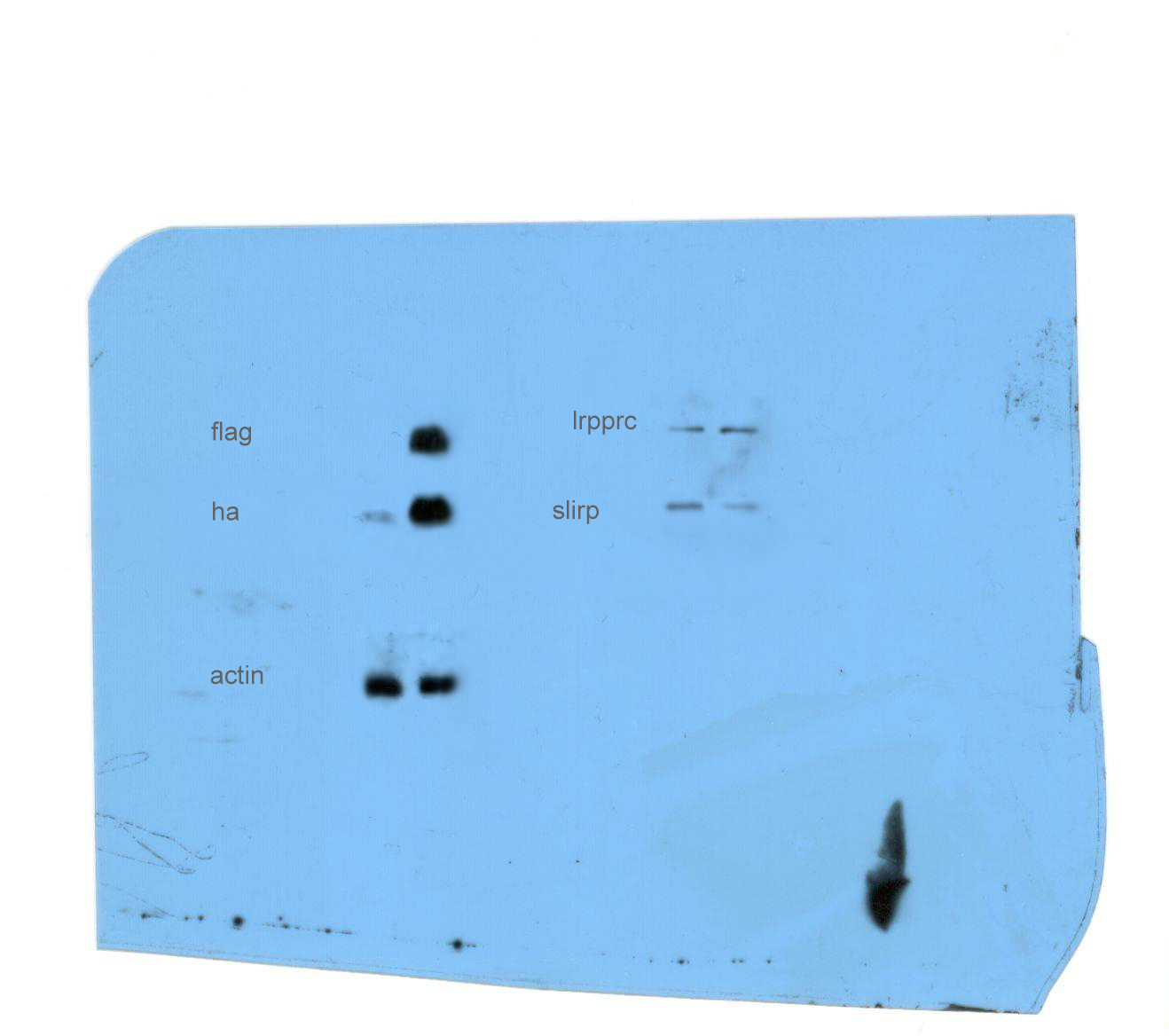

Supplement: S1 File — (ZIP) [file pone.0238857.s001.zip › original uncropped and unadjusted images/figure 7c/7c.jpg]

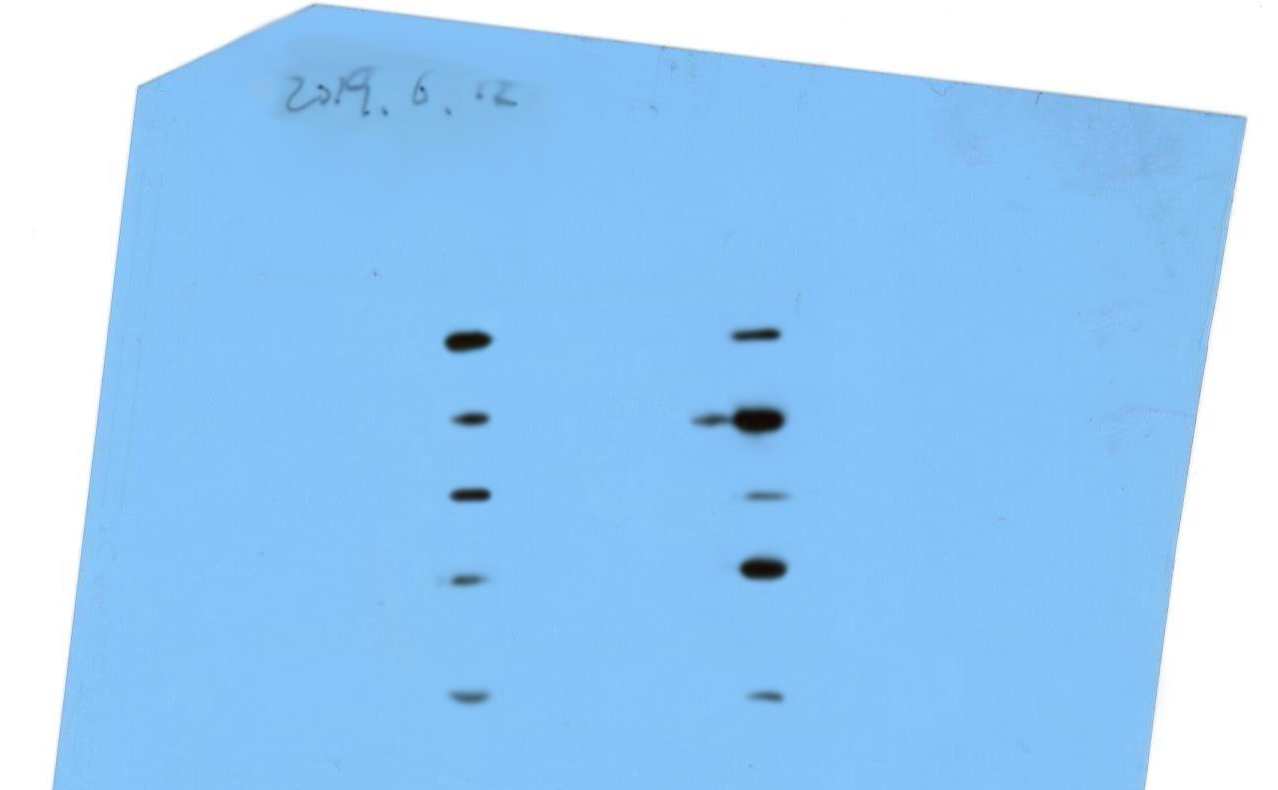

Supplement: S1 File — (ZIP) [file pone.0238857.s001.zip › original uncropped and unadjusted images/figure 7c/x file-3 2019.6.130001.jpg]
