# Supplementary material for: Propofol induces mitochondrial-associated protein LRPPRC and protects mitochondria against hypoxia in cardiac cells
Source: PLoS One. 2020 Sep 8;15(9):e0238857. doi: 10.1371/journal.pone.0238857 (PMC7478836; doi:10.1371/journal.pone.0238857)
Supplement: Attachment — Submitted filename: respond to reviewers.docx [file pone.0238857.s002.docx]

Reviewer #1: In the current study “Mitochondrial-associated protein LRPPRC is critical in propofol-induced protective effect against oxidative stress in cardiac cells” the authors discussed the involvement of LRPPRC in propoofl-induced cardiac cell protection in hypoxia. Here are some major and minor concerns:
Major:
1. In Figure 1A, why would 48h normoxia group drop in cell viability comparable to the hypoxia group? Does it mean hypoxia is not the reason of cell death?

Answer： Dear editor, thanks for the notification. In this section, Normoxia condition with supplemented CoCl2, which is a hypoxia mimic, was considered as a control of hypoxia. By comparing to normoixa+CoCl2 group, the decrease in cell viability by hypoxia exposure was considered to be induced by upregulation of HIF-1a. The figure has been modified.

2. In Figure 1C, pro-caspase-9 and cleaved caspase-9 image is not consistent with statistics.

Answer： Sorry for the mistake. In figure 1C, we incorrectly present the statistical data of image.

3. In Figure 2A, without propofol, 24h hypoxia reduces LRPPRC protein expression compared with normoxia group. However, this trend does not show in Figure 2D and many other subsequent figures. The authors need to explain the discrepancy.

Answer： Thanks for the precious suggestion. According to our results, in figure 2A and C, 24-hour exposure to hypoxia decreased LRPPRC. In figure 2D, 48-hour exposure to hypoxia decreased LRPPRC, but not at 24-hour time point. To figure out the discrepancy, we detected LRPPRC protein level at different time points, including 12, 16, 20, 24, 28, 32, 36, 40, 44, 48h in normoxia and hypoxia group, repeatedly. As shown in our supplemented results (Figure 1), LRPPRC was obviously decreased at 28, 32, 36, 40, and 48 h. However, at 24-hour time point, LRPPRC was not obviously decreased. This instability may explain at 24-h time point after hypoxia exposure, the observation of decrease in LRPPRC was not consistent. That’s why we mainly focused on the 48-hour time point.

Figure 1. After 12, 16, 20, 24, 28, 32, 36, 40, 44 and 48-hour hypoxia exposure,

4. In Figure 6A, why would hypoxia mock treatment be so different from hypoxia siNC treatment? The increase of LC3-II in hypoxia condition compared to normoxia in Figure 5E is also absent in Figure 6A.

Answer：Dear editor, we found that transfection procedure obviously upregulated LRPPRC. This may due to a technical character. If you think it is unacceptable, we’d like to change another technic to introduce siRNA.

5. The authors found a direct binding between LRPPRC and Bcl-2, and the two protein expression is the same trend. However, it is not sufficient enough to claim that LRPPRC stabilizes Bcl-2. At least the authors need to detect the mRNA Bcl-2, and use translational inhibitors as well as proteasome inhibitors combined with the knockdown of LRPPRC to make a conclusion.

Answer： The stabilizing activity of binding of LRRPRC to Bcl-2 was reported previously (1). We would like to claim that upregulated LRPPRC may function via stabilizes Bcl-2.

Zou J, Yue F, Jiang X, Li W, Yi J, et al. (2013) Mitochondrion-associated protein LRPPRC suppresses the initiation of basal levels of autophagy via enhancing Bcl-2 stability. Biochem J 454: 447-457.

6. Figure 8C right, why would LRPPRC and SLIRP blots not detect the overexpression of these genes?

Answer：Dear editor, in mammal cells, the overexpression of introduced coding sequence is usually not so obvious because of relative low introducing efficacy. The flag- or HA-tagged protein was usually obvious in IP product because of immune enrichment.

7. In Figure 8, results claim LRPPRC stabilizes SLIRP, instead of SLIRP stabilizes LRPPRC. This is the opposite of regulation mechanism to the published articles. The authors need to point it out and give a thorough discussion.

Answer： Thanks for the suggestion, we also agree with that it is not sufficient to clain LRPPRC stabilizes SLIRP. According to our results in figure 8, we presented the evidence showing that LRPPRC binds to SLIRP only. So we modified the manuscript.

8. It is best the authors detect the endogenous SLIRP and LRPPRC binding, because the overexpression system sometimes introduce artificial results due to the expression abundance and overexpression techniques.

Answer：We previously detected endogenous LRPPRC and SLIRP. However, we failed to intensively detect the binding between endogenous LRPPRC and SLIRP. The potential reasons are listed as followed: (1) antibody target to endogenous LRPPRC and SLIRP are not suitable for immunoprecipitation analysis because of low efficacy. (2) The low endogenous protein level of LRPPRC or SLIRP.

After several failure, we have to decide to introduce exogenous LRPPRC and SLIRP with Flag or HA tag.

Minor:
9. There is no evidence to “Bcl-2 binds and stabilizes the SLIRP protein and thus enhances its function” in the discussion. The authors should be careful in conclusions.

Answer： Thanks for the suggestion, it has been modified in 4 th paragraph of discussion section.

10. Legend of Figure 4 is missing.

Answer： Sorry for the mistake!

11. Instead of LRPPRC protects the mitochondria from autophagy, it is more likely that with LRPPRC, mitochondria are not damaged so that they do not have to be eliminated by autophagy (membrane potential and function is rescued). The authors need to revise the manuscript.

Answer： Thanks for the suggestion. We agree with this precious suggestion.

12. It is best the authors put the same genes into groups and distinguish with experimental conditions in the group graphs.

Answer： Do you mean the figure 2A, protein levels?

13. The authors never discussed oxidative stress by analyzing ROS or the ROS eliminating mechanisms. The model used is hypoxia and there may be a lot of mechanisms in cytotoxicity. Plus, oxidative stress is best manifested in the reperfusion phase which the authors did not study. So it is best the authors correct the title and related areas from oxidative stress by focusing on hypoxia.
Answer： Thanks for the suggestion. We modified the title and manuscript accordingly.

Reviewer #2: The manuscript entitled “Mitochondrial-associated protein LRPPRC is critical in propofol-induced protective effect against oxidative stress in cardiac cells” suggested the protective effects of propofol on hypoxia induced cell injury on H9C2 cells and rat cardiac myocytes via LRPPRC-SLIRP-Bcl2 regulation. In detail, propofol treatment inhibited hypoxia induced apoptosis and autophagy in H9C2 cells. Authors further demonstrated that LRPPRC, SLIRP and Bcl-2 potentially form heterotrimers using immunoprecipitation. Based on accumulated results, the authors clearly described the mechanism of propofol on regulation of mitochondrial homeostasis under hypoxia conditions. However, there are several concerns must be addressed.

1) To support authors’ idea that anti-oxidative stress effects of propofol, oxidative stress markers such as 4HNE or MDA should be checked.

Answer： Thanks for the precious suggestion. Instead of 4HNE or MDA, we measured the ROS accumulation, which is also considered as a major cause of oxidative stress. Moreover, we analyzed cell viability and apoptosis as a presentation of oxidative stress-related cell injury. 4HNE and MDA were often employed in patient serum who is suffering oxidative stress-related disease.

2) In Fig1A, cell viability of normoxia condition at 48h is significantly decreased similar to that of hypoxia condition. Please check the data and if that is correct please discuss it whether that result is related to unchanged expression of LRPPRC with hypoxia induction compared to normoxia group (Fig 2 B, C).

Answer： Dear editor, thanks for the notification. In this section, Normoxia condition with supplemented CoCl2, which is a hypoxia mimic, was considered as a control of hypoxia. By comparing to normoixa+CoCl2 group, the decerase in cell viability by hypoxia exposure was considered to be induced by upregulation of HIF-1a. The figure has been modified.

3) For the western blot analysis, please include quantification results. For Fig 1C and 2A, what do the apoptotic death rate graphs mean?

Answer： Sorry for the incorrect labeling. They have been modified.

4) For Fig 2A & B, please include normoxia 48h group for control.
Answer： The control group was originally included.

5) For Fig 3A, please include HIF-1a western results. The asterisk (*) on results of DFO and CoCl2 treatment groups seems be missing, considering the mention on p16.

Answer： Thanks for the remaindering. The missing part has been added.

6) For Fig 4C, please describe the reason of unchanged HIF-1a protein expression with echinomycin treatment. Please consider for checking HIF-1a expression from nuclear fraction.

Answer： Echinomycin was considered as a inhibitor of HIF-1a’s transcriptional activity. It was proved that echinomycin do not affect HIF-1a protein level.

7) For Fig 6, data from mock group is confusing. It should be similar to that of siNC.

Dear editor, we found that transfection procedure obviously upregulated LRPPRC. This may due to a technical character. If you think it is unacceptable, we’d like to change another technic to introduce siRNA.

8) For Fig 6, it should be checked whether maintaining mitochondrial homeostasis effects of propofol against hypoxic conditions was blocked with siLRPPRC transfection.

Answer： Introduction of siNC obviously upregulated LRPRC, which is unexpected. Knockdown of LRPPRC by siLRPPRC introduction obviously decreased mitochondrial mass.

9) In general, there are some errors/mistakes in the figures. Please re-check them carefully and thoroughly. Some identified was shown below.
- For AnnexinV/PI assay results, indications seem to be incorrect.
Fig 1B & 3C: horizontal: PI/ vertical: Annexin V-> horizontal: Annenxin V/ vertical: PI
Fig4D: missing-> horizontal :PI , vertical : PI
- Fig 4C, please check the indication for propofol treatment +/-.

Answer： Thanks for these suggestions. All figures have been re-checked.

10) Materials and methods part contains less information.
- Please describe how hypoxia condition was induced on cell cultures.
- Please include methods for siLRPPRC transfection.
- Please add methods for caspase3/9 activity measurements and immunoprecipitation.

Answer： Thanks for these suggestions. It has been re-checked.
