# Supplementary material for: Propofol induces mitochondrial-associated protein LRPPRC and protects mitochondria against hypoxia in cardiac cells
Source: PLoS One. 2020 Sep 8;15(9):e0238857. doi: 10.1371/journal.pone.0238857 (PMC7478836; doi:10.1371/journal.pone.0238857)
Supplement: Attachment — Submitted filename: response to reviewers.docx [file pone.0238857.s003.docx]

Reviewer #1: In the present version of “Propofol induces Mitochondrial-associated protein LRPPRC and protectives mitochondria against oxidative stress in cardiac cells”, the authors made a lot of explanation, correction and improvement. Here are some more minor concerns to be addressed:
1. If Figure 1A is the only experiment to supplement CoCl2 to mimic hypoxia but called control or normoxia, it is best not to show the CoCl2 treatment results. Instead, a non-treated group should be shown to make the whole study consistent. CoCl2 does not provide any more information, but confusion to the study.

Answer: Thanks for the suggestion. Originally, we already performed normoxia group without cocl2. We’d like to replace normoxia+cocl2 group with normoxia group.
2. If LRPPRC expression is modified by siRNA transfection, the authors should mention it in the result or discussion parts to reduce confusion.

Answer: It has been mentioned in discussion parts.
3. Although the authors changed the title, oxidative stress is still in it. If the authors insist on claiming oxidative stress, ROS detection should be added and the reperfusion phase should be studied. The source of ROS and ROS elimination machinery should also be analyzed. The easier way is to correct oxidative into hypoxia, which is what the authors studied in the present research project. Also, is it "protectives" or "protects"?

Answer: Thanks for the suggestion. We further modified the title.
4. For Figure 1C, Figure 2A, the authors should put genes in groups and assign different colors to different experimental condition, the same way of Figure 6D.

Answer: Thanks for the suggestion. We further modified the figures.
5. The authors need to double check the Annexin V/PI staining original figures. It seems in Figure 1 and 3, all the axes for Annexin V is labeled PI and vice versa.

Answer: Thanks for the suggestion. We further modified the figures.
